# Morphologies, Compatibilization and Properties of Immiscible PLA-Based Blends with Engineering Polymers: An Overview of Recent Works

**DOI:** 10.3390/polym16131776

**Published:** 2024-06-23

**Authors:** Amulya Raj, Mohamed Yousfi, Kalappa Prashantha, Cédric Samuel

**Affiliations:** 1 IMT Nord Europe, Institut Mines-Télécom, Univ. Lille, Centre for Materials and Processes, 59000 Lille, France; prashantha.kalappa@gmail.com (K.P.); cedric.samuel@imt-nord-europe.fr (C.S.); 2 Université de Lyon, CNRS, UMR 5223, Ingénierie des Matériaux Polymères, Université Claude Bernard Lyon 1, INSA Lyon, Université Jean Monnet, 69621 Villeurbanne Cedex, France; 3 ACU-Centre for Research and Innovation, Faculty of Natural Sciences, Adichunchanagiri University, B.G. Nagara, Mandya 571448, Karnataka, India

**Keywords:** Poly(L-Lactide), polymer blends, engineering polymers, compatibilization, durable and high-performance applications

## Abstract

Poly(L-Lactide) (PLA), a fully biobased aliphatic polyester, has attracted significant attention in the last decade due to its exceptional set of properties, such as high tensile modulus/strength, biocompatibility, (bio)degradability in various media, easy recyclability and good melt-state processability by the conventional processes of the plastic/textile industry. Blending PLA with other polymers represents one of the most cost-effective and efficient approaches to develop a next-generation of PLA-based materials with superior properties. In particular, intensive research has been carried out on PLA-based blends with engineering polymers such as polycarbonate (PC), poly(ethylene terephthalate) (PET), poly(butylene terephthalate) (PBT) and various polyamides (PA). This overview, consequently, aims to gather recent works over the last 10 years on these immiscible PLA-based blends processed by melt extrusion, such as twin screw compounding. Furthermore, for a better scientific understanding of various ultimate properties, processing by internal mixers has also been ventured. A specific emphasis on blend morphologies, compatibilization strategies and final (thermo)mechanical properties (tensile/impact strength, ductility and heat deflection temperature) for potential durable and high-performance applications, such as electronic parts (3C parts, electronic cases) to replace PC/ABS blends, has been made.

## 1. Introduction: A Quest for High-Performance PLA-Based Materials

**Figure 1:**
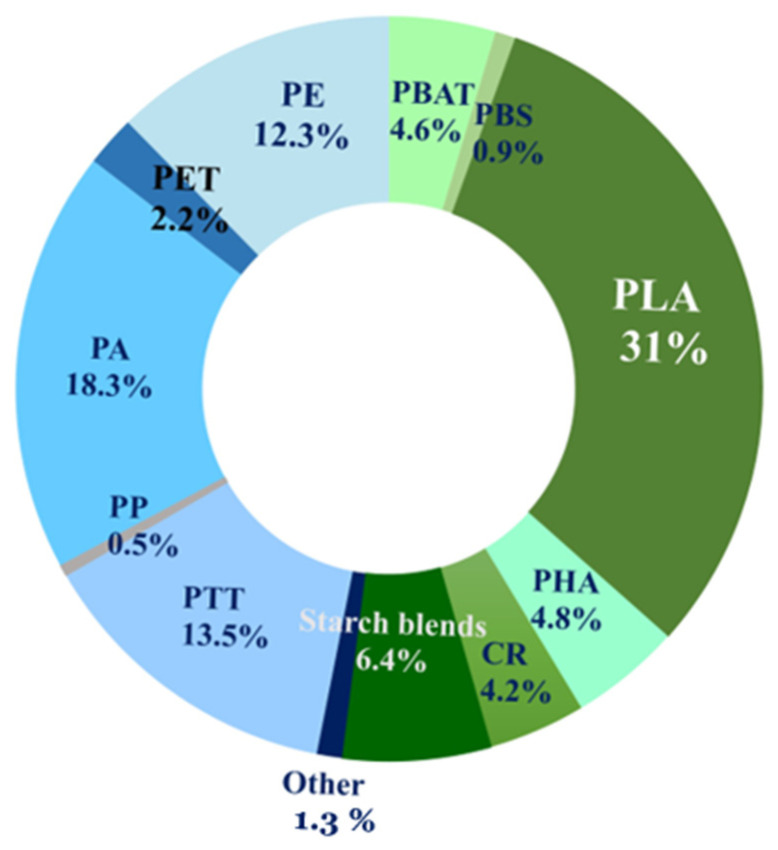
Global production of biobased and/or biodegradable plastics in 2023. Reproduced/adapted from [1].

The production of biobased polymers from sustainable resources has exponentially increased in the last decade due to growing environmental concerns related to classical petroleum-based polymers. According to the European Bioplastics association, nearly 2.18 million tons of biobased polymers were produced in 2023, and biobased polymers currently account for nearly 0.5% of the 400 million tons of polymers produced every year [1]. The market of biobased polymers is increasing by 20–30% each year and poly(L-Lactide) (PLA) currently holds a significant part of this market, close to 31% (Figure 1) [1]. PLA was discovered in 1932, but this material could be still considered as an emerging biobased polymer because efficient and cost-effective routes to high-molecular weight PLA were only recently set up at the industrial scale. The major method to produce high-molecular weight PLA is based on the ring-opening polymerization of lactides, with such monomers being obtained by the fermentation of corn, beet and cane sugar [2,3,4,5,6]_._

PLA has gained significant attention due to various interesting properties, such as high stiffness/strength (tensile modulus ≈ 3–4 GPa and tensile strength ≈ 50–60 MPa), biocompatibility/biodegradability and good processability by melt-state extrusion processes [3,7,8,9]. Another advantage is linked to the cost of PLA that has significantly dropped over the past 10 years to 3–5 EUR/kg due to an increased industrial maturity. Important applications are consequently found in medicine (sutures, surgical implants, and drug delivery systems) [3,6,10] and intensive research is also performed in tissue engineering to develop scaffolds [11,12]. Packaging is another important application of PLA [13,14,15], and PLA-based containers for water, juice and yogurt are currently used in Europe, Japan and North America [13,15,16]. The application of PLA-based materials extends to waste-composting bags, mulch films and controlled release matrices for fertilizers, pesticides and herbicides [5,17]. PLA is also suitable to produce filaments and fabrics for the textile industry [18,19]. Finally, over the past few years, PLA is being used in niche applications such as shape-memory materials [20,21], 3D-printing [22,23] and piezoelectric devices [24].

However, PLA has some shortcomings, such as low ductility (strain at break < 10%), poor impact strength (<5–25 kJ/m^2^) and low thermal resistance (HDT, heat deflection temperature close to glass transition temperature ≈ 60 °C) [2,3,5,7,8,9,25,26]. These limitations hinder the use of PLA in durable and high-performance applications, such as structural applications for transportation/electronic markets, requiring long-term stability over a broad range of thermomechanical conditions (in particular at elevated temperatures up to 100–130 °C). Various strategies have been proposed to overcome these drawbacks, including the incorporation of plasticizers, impact modifiers, nucleation agents, (nano)fillers and the use of PLA stereocomplexes. Several comprehensive reviews have been provided earlier on these topics [6,9,27,28,29,30,31]. Plasticizers and impact modifiers could significantly improve the ductility and the impact strength of PLA. For example, citrate esters (such as triethyl citrate and tributyl citrate) [32,33,34] and poly(ethylene glycol) [9,34] are particularly efficient for this purpose. Concentrations higher than 10–20% are required with such plasticizers. Numerous impact modifiers have been also proposed to improve PLA impact strength and, for example, poly(ether-*block*-amide), at a concentration close to 30%, significantly improves the impact strength of PLA [9]. Random biodegradable copolyesters, such as P[CL-co-LA] or P[CL-co-VL], have been also successfully tested as efficient impact modifiers [30,35]. However, plasticizers and impact modifiers reduce the tensile strength of PLA and, more importantly, these additives do not significantly improve the thermal resistance of PLA. Consequently, plasticizers and impact modifiers cannot be considered alone for durable and high-performance applications requiring high-temperature stability. The incorporation of nucleating agents and/or rigid (nano)fillers is quite interesting, to enhance the crystallinity and the HDT of PLA without the reduction of the tensile modulus/strength. Numerous inorganic and organic agents are cited in this field, such as talc, calcium carbonate, halloysites, montmorillonites, titanium dioxide, graphite, carbon nanotubes, graphene, cellulose nanocrystals, *N*,*N*′-ethylene bis-stearamide and hydrazide compounds [36,37,38,39,40]. Recently, the association of PLA with its enantiomeric counterpart poly(*D*-Lactide) (PDLA) also led to significant improvements in terms of PLA crystallinity due to the formation of peculiar PLA stereocomplexes [41]. High amounts of PLA stereocomplexes displaying high melting temperatures ranging from 200–230 °C (i.e., 30–60 °C higher than PLA homocrystals) could be obtained in equimolar associations. In this context, the use of PLA stereocomplexes represents a promising approach for future PLA-based materials [41,42]. However, nucleating agents, rigid (nano)fillers and PLA stereocomplexes do not efficiently improve the ductility and the impact strength of PLA. Thus, these approaches cannot be also considered alone for durable and high-performance applications. In order to obtain a perfect balance between stiffness—ductility—impact strength—HDT, mixed/combined approaches are required [40,43,44,45] and complex PLA-based formulations now enter the market. Although these mixed/combined strategies could be proposed by various manufacturers at the semi-industrial scale, economical and sustainability issues still remain a pending question regarding these complex formulations, incorporating multiple (non-biobased) additives that considerably increase processing/material costs without clear environmental benefits.

Polymer blending is yet another approach to improve the shortcomings of PLA and this route is quite attractive due various positive attributes. First, PLA-based blends with engineering polymers displaying high thermomechanical properties could be viewed as a balanced approach between previously discussed approaches based on impact modifiers and rigid (nano)fillers. A full set of thermomechanical properties could theoretically be reached with these PLA-based blends, in particular higher ductility, impact strength and HDT without the reduction in the tensile modulus and strength [46,47]. Then, polymer blending is recognized as a cost-effective approach with a high ease of commercialization due to solvent-free processing using melt-state extrusion technologies. In this context, PLA blending with poly(carbonate) (PC), poly(ethylene terephthalate) (PET), poly(butylene terephthalate) (PBT) and various poly(amides) (PA) are currently being extensively investigated for advanced applications. It could be mentioned that these PLA-based blends could also bring about important options with the use of recycled PET/PBT materials and (partly) biobased PC/PA materials. The morphologies and compatibilization of these PLA-based blends play a key role on final properties. Hence, the focus of the present overview is to comprehensively review recent advances in PLA-based blends with engineering polymers (such as PC, PET, PBT and PA) to improve its ductility, impact strength and the HDT of PLA for durable and high-performance applications. A particular focus is provided on blend morphology, compatibilization strategies and final thermomechanical properties.

## 2. Fundamental Concepts Regarding Polymer Blends

A polymer blend is defined as a combination of two or more polymers with a concentration higher than 2 vol.%, and a plethora of polymer blends have been developed over the past 50 years, in particular by twin-screw extrusion processing in the melt state [46,48]. Essentially, polymer blending technologies clearly represent cost-effective and industrially relevant solutions to reach high-performance polymer materials with a precise tuning over multiple final properties [46,47]. PLA-based blends have been extensively considered in the last decade to tackle the various drawbacks of these biobased materials for a deeper market penetration into advanced applications. Before depicting PLA-based blends with engineering polymers, it is of importance to uncover some fundamental concepts related to polymer blends.

### 2.1. Miscible Polymer Blends

Miscibility in polymer blends is the first aspect to consider, with a basic classification between miscible and immiscible polymer blends. Miscible polymer blends represent interesting case studies with high practical interests. These blends exhibit a single-phase morphology arising from an intimate mixing of the polymers at the macromolecular level, and final properties generally follow an additivity rule according to the volume ratio of each polymer [21,47,49]. A very fine tuning over multiple properties is offered, and the miscibility generally arises from specific interactions leading to favorable enthalpic contributions on the Gibbs free energy of mixing [47].

### 2.2. Immiscible Polymer Blends—Morphology

Most of the polymer blends are based on commercially available polymers; the above mentioned criterion is rarely fulfilled due to unfavorable interactions, leading to immiscible polymer blends with dual-phase morphologies [47]. However, interesting final properties are also depicted for immiscible polymer blends with potential synergistic effects, although a careful control over the blend morphology is required.

**Figure 2:**
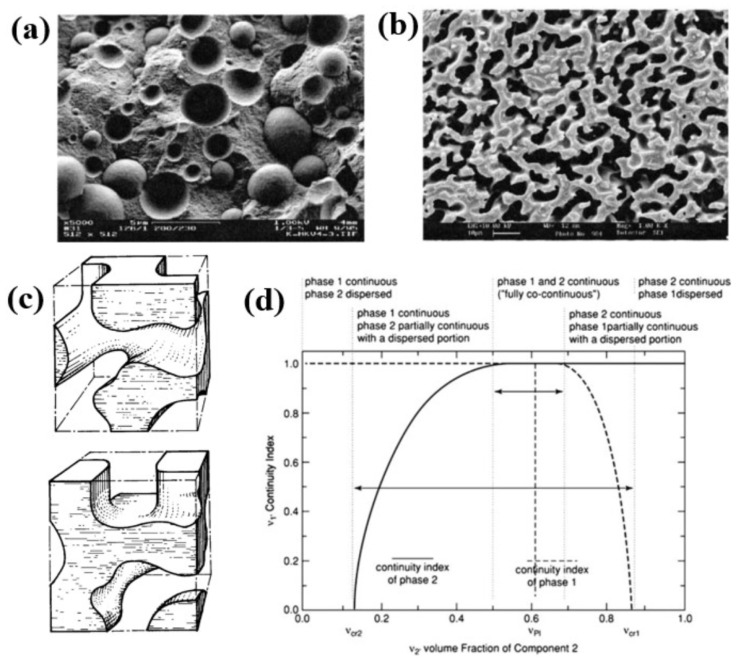
Typical matrix/droplet morphology (TPU/PP 80/20) (**a**) and co-continuous morphologies (PE/PS 25/75) (**b**) obtained for immiscible polymer blends. Reproduced/adapted from [46]. Morphology model of both components in a co-continuous blend (**c**). Reproduced/adapted from [46]. Typical continuity diagrams obtained for immiscible polymers with the transition between matrix/droplet and co-continuous morphologies (**d**). Reproduced/adapted from [47]. Continuity index is the fraction of the phase that is continuous through the sample. Dispersed droplets have a continuity index of 0, while completely continuous phase have a degree of 1. v_cr1_, v_cr2_, v_f1_ and v_f2_ designate fractions in the volume of phase 1 or 2, at which the full or partial co-continuity of the related phase starts. v_PI_ represents phase inversion composition.

Two main types of dual-phase morphologies are attested for immiscible polymer blends (Figure 2a,b). Matrix/droplet morphologies are described as a dispersion of spherical droplets (also called nodules) of a polymer B in a continuous matrix of a polymer A, and these morphologies are often observed for moderate concentrations (typical range approx. 0–30 vol.%). Co-continuous morphologies are depicted by an interpenetrated network of the two polymers (Figure 2c) and appear for higher concentrations (approx. range 30–50 vol.%). Continuity diagrams are of good importance to visualize the transition between matrix/droplet and co-continuous morphologies with respect to volume ratio (Figure 2d).

**Figure 3:**
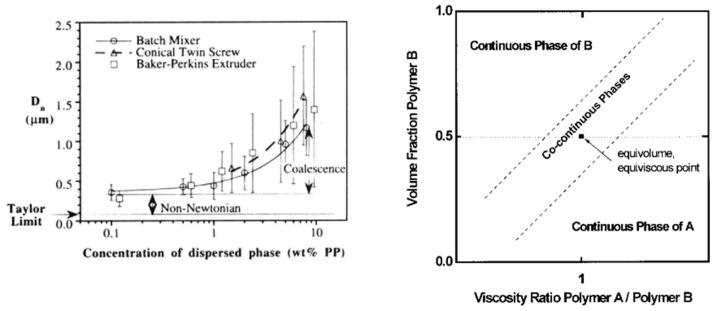
(**Left**) Number-average droplet diameter (D_n_) of polypropylene in a polystyrene matrix (viscosities are, respectively, 950 Pa.s and 840 Pa·s at 200 °C and 65 s^−1^). Results are displayed for various processing techniques, i.e., batch mixer and twin-screw extruders. The Taylor limit is given that corresponds to R_lim_ according to Equation (2). Reproduced from [52]. (**Right**) Typical co-continuous window as a function of the volume fraction of polymer B and the viscosity ratio. Reproduced from [46].

Matrix/droplet morphologies are particularly interesting for ductility/tensile—impact strength improvements, and a careful attention to the droplet size/shape is required. The final droplet size is governed by an equilibrium between droplet breakup and coalescence mechanisms. Flow conditions imposed during processing (shear/elongational rate, temperature, etc.) and the rheological/interfacial characteristics of each phase play a key role on these mechanisms [50]. The capillary number *C_a_* (Equation (1)) is a fundamental parameter of the droplet breakup mechanism that represents the ratio between (i) the stress imposed by the matrix that deforms the droplet (term $\eta_{m}\cdot \dot{\gamma }$) and (ii) interfacial forces that maintain droplets into a spherical shape (term σ/R). Droplet breakup occurs for critical values [51] and, for blends of Newtonian fluids with a viscosity ratio smaller than 2.5 and low droplet concentrations (typically < 5 vol.%), the droplet size theoretically tends to a limit value (R_lim_) according to the equation (Equations (2) and (3)) [51,52]. However, polydisperse droplets with higher sizes are always observed for blends of viscoelastic polymers, due to non-Newtonian effects and droplet coalescence phenomena (Figure 3). Droplet coalescence represents a major phenomenon, even for concentrations as low as 5 vol.%, and deformation rate/time plays a key role on the droplet breakup—coalescence equilibrium. It could be finally mentioned that viscoelastic properties also have a profound effect on the final shape of the dispersed phase. Fibrillar morphologies (i.e., dispersed phase with a high aspect ratio) could be generated for elevated C_a_ and matrix elasticity [53]. Stable (nano)fibrillar morphologies are evidenced for specific polymer blends processed at high shear rates with obvious improvements of the final (thermo)mechanical properties [53].

(1) $$C_{a}=\frac{\eta_{m}\cdot \dot{\gamma }\cdot R}{\sigma }$$

(2) $$R_{lim}=\frac{8\cdot \sigma \cdot \left(p+1\right)}{\eta_{m}\cdot \dot{\gamma }\cdot \left(19p+16\right)}$$

(3) $$p=\frac{\eta_{d}}{\eta_{m}}$$

where $\eta_{m}$ the matrix viscosity, $\eta_{d}$ the dispersed phase viscosity, $\dot{\gamma }$ the shear rate imposed, R the droplet radius, $R_{lim}$ the droplet radius at equilibrium in a shear flow, σ the interfacial tension, p the viscosity ratio.

The percolation of the minor phase into continuous structures could occur for higher concentrations, giving rise to co-continuous morphologies. Ductility/impact strength improvements are attested, but these morphologies also offer efficient combinations of all physico-chemical properties [46,47]. In particular, enhanced heat deflection temperatures could be obtained with continuous structures of an engineering polymer into commodity matrices [46,47,54,55,56,57]. The main challenge lies in the generation of stable co-continuous morphologies with controlled morphological features by melt-state processing. The appearance of co-continuous morphologies mainly depends on the volume fraction and viscosity ratio, with typical co-continuity windows depicted in Figure 3. However, co-continuous morphologies tend to be unstable during melt-state processing and transform into matrix/droplet morphology at the equilibrium due to phase inversion considerations, according to Equation (4) [46,58]. Thus, co-continuous morphologies only appear during the intermediate stages of the mixing/phase inversion. In this respect, the development of co-continuous morphologies requires an advanced control over processing conditions and the rheological/interfacial features of each phase.

(4) $$\phi_{PI,2}=\frac{1}{1+p}$$

where $\phi_{PI,2}$ the concentration of the polymer 2 for phase inversion.

### 2.3. Compatibility in Immiscible Polymer Blends

**Figure 4:**
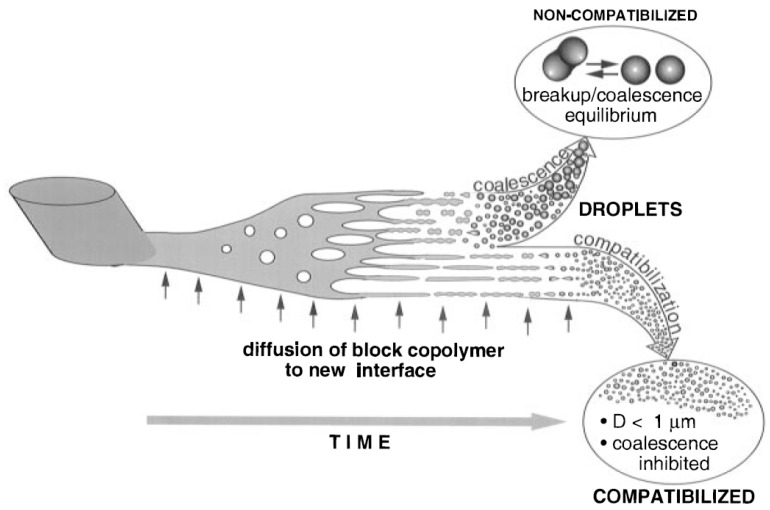
Morphology development pathways during melt blending of non-compatibilized and compatibilized polymer blends. Reproduced from [63].

A final important aspect of immiscible polymer blends lies in interfacial effects and compatibilization strategies. The interface quality critically influences the final properties of the blend [47], and the interfacial tension represents an important parameter to control various aspects linked to the compatibility of immiscible polymer blends. The interfacial tension plays a key role on droplet breakup (according to Equations (1) and (3)), coalescence and interdiffusion mechanisms. Thus, polymer blends that display favorable interfacial tensions tend to generate refined and homogenous morphologies [47,59,60]. Enhanced interfacial thicknesses are also observed due to interdiffusion mechanisms, up to the generation of a significant interphase in specific blends with ultra-low interfacial tensions [47,48,61]. The level of interfacial adhesion in the solid state is also partly linked to interfacial tension [47,61]. Polymer blends with favorable interfacial tensions tend to display higher interfacial adhesion that enable efficient stress transfers between blend components. These aspects give rise to compatible blends with potential synergistic effects in terms of final properties, in particular on final (thermo)mechanical properties [47]. However, most of the commercial polymer blends clearly display a high level of immiscibility and the use of compatibilizers is often mandatory. Classical approaches include the use of block copolymers or reactive copolymers [47,62,63]. Block copolymers with a selective solubility in each blend component are required to insure their location at the blend interface [47,63,64,65,66,67], but reactive copolymers are more versatile and industrially relevant for numerous polymer blends. Specific functionalities (isocyanate, epoxy, anhydride or other chemical groups) induce specific reactions with one (or several) blend component(s) inducing an in situ production of the effective compatibilizer during melt-state processing [47,63,68]. In each case, an emulsification effect is achieved that drastically reduces interfacial tensions between blend components, inducing refined and homogeneous morphologies according to the droplet breakup mechanism (Equations (1) and (3)). An efficient suppression of the coalescence phenomenon is also claimed to explain the high efficiency of compatibilizers (Figure 4) [67,68,69,70]. Thick and strong interphases are also attested in these compatibilized blends [47,48,61], with the obvious enhancement of the entire final properties.

## 3. Overview of PLA-Based Blends with Engineering Polymers

### 3.1. General Considerations

PLA-based blends have gained considerable attention in the scientific/industrial communities, as attested by the surplus amount of research articles published on PLA-based blends within the last decade. Numerous commercial polymers have been blended with PLA depending on the end-use applications, and PLA-based blends could be classified into several categories. A large focus was placed on PLA-based blends with biodegradable and (partly)biobased polymers. The main partners for PLA in this field include thermoplastic starch [71], poly(ε-caprolactone) (PCL) [72], poly(hydroxy alkanoates) (PHA) [73], poly(butylene succinate) (PBS) [74], poly(butylene succinate-*co*-adipate) (PBSA) [75] and poly(butylene adipate-*co*-terephthalate) (PBAT) [76]. To make PLA suitable for food or cosmetic packaging fields, blending with other biopolymers, such as poly(hydroxybutyrate) (PHB) and poly(3-hydroxybutyrate-*co*-3-hydroxyvalerate) (PHVB), is considered to be an easy and cost-effective strategy [77,78]. The state-of-the-art of this aspect will not be covered in the present review, since it requires a specific dedicated article.

PLA-based blends with commodity plastics such as low-density polyethylene (LDPE) [79], high-density polyethylene (HDPE) [80], polypropylene (PP) [81] and polystyrene (PS) [82] were also considered with limited applicative potentials. Rubbers, elastomers and elastomer-like materials, such as natural rubber (NR) [83], isoprene rubber (IR) [84], styrene butadiene styrene rubber (SBS) [85] and thermoplastic polyurethanes (TPU) [86], are also regularly mentioned as valuable partners, in particular to improve the ductility/impact strength of PLA. However, all these strategies reduce the tensile properties of PLA and fail to enhance the heat deflection temperature of PLA.

**Figure 5:**
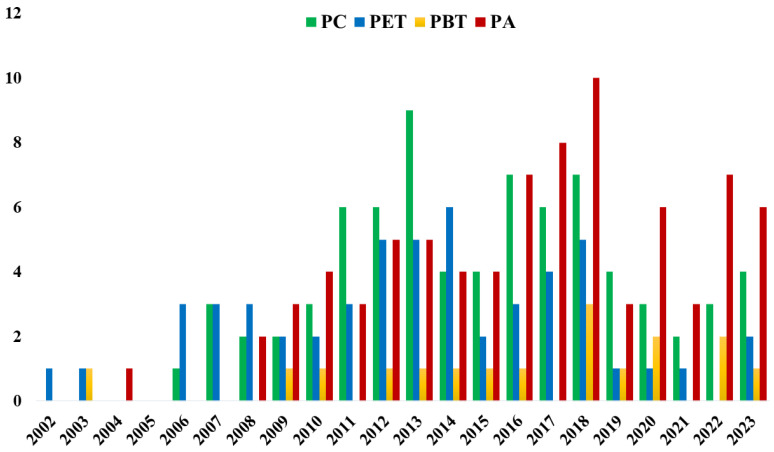
Yearly evolution of the amount of research articles on PLA-based with engineering polymers since 2002.

These shortcomings could be theoretically tackled using PLA-based blends with engineering polymers, such as poly(methyl methacrylate) (PMMA), polyamides (PA), polycarbonate (PC), poly(ethylene terephthalate) (PET) and poly(butylene terephthalate) (PBT). The yearly evolution of the research articles on PLA-based blends with these engineering polymers is roughly displayed in Figure 5. During the period 2010–2019, an approximative number of 30 research articles per year is attested on this topic and various aspects are covered, including formulation, processing, morphologies, crystallization and final properties. In this context, this overview aims to gather important results in the field of PLA-based with engineering polymers, in particular for immiscible PLA-based blends with PC, PET, PBT and PA that displayed dual-phase morphologies. The case of PLA/PMMA blends will be not treated herein, because these blends usually displayed single-phase morphologies arising from PLLA/PMMA miscibility [21,49,87,88].

**Figure 6:**
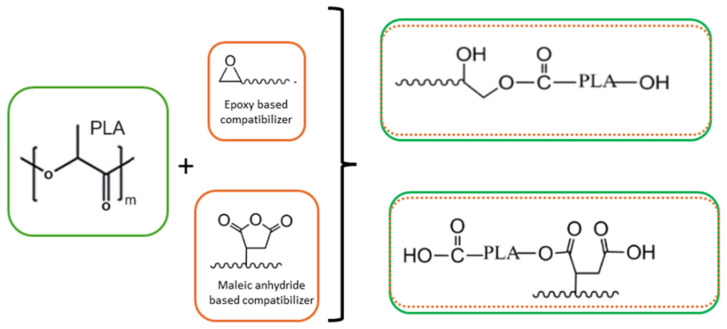
Compatibilization strategies of PLA-based blends with PC, PET/PBT and PA.

Table 1 gathers the standard thermomechanical properties observed for neat PLA, PC, PET and PBT. This table is built using various available technical data sheets given by emblematic producers, i.e., NatureWorks/Total-Corbion for PLA Ingeo^®^/PLA Luminy^®^ grades, respectively, Covestro for PC Makrolon^®^ grades and DSM for PET/PBT Arnite^®^ grades. The case of PA will be discussed in a specific section, as various types of PA have been explored for blending with PLA. PLA clearly displays an outstanding tensile modulus (3.4–3.7 GPa) with a high tensile strength of comparable magnitude to PC, PET and PBT. In this context, PLA-based blends with engineering polymers could be processed easily by twin screw extrusion. The blends could theoretically display high tensile modulus in the range 2.5–3.5 GPa and high tensile strength superior to 60 MPa. Ductility, impact strength and HDT are significantly higher for all engineering polymers than PLA, due to the high glass transition temperature (for PC) and/or high crystallinities (for PET/PBT). In this context, blending PLA with these engineering polymers could provide new materials with higher ductility, impact strength and thermal resistance for durable and high-performance applications. Compatibilizers are generally required to improve the interface between these blends. General compatibilizers used to improve the interface between PLA, and other engineering polymers are maleic based or anhydride based. In some cases, even the formation of copolymers by transesterification is also probable. A general schematic of the compatibilization strategy is depicted in Figure 6. In all cases, formulation parameters (type of polymer, blend composition, additives, etc.) and processing parameters will pay a crucial role on final properties. The impact of these parameters is discussed in the following sections.

### 3.2. PLA-Based Blends with Poly(Carbonate) (PC)

Poly(carbonate) (PC), or more precisely poly(bisphenol-A carbonate), clearly represents one of the most efficient engineering thermoplastic polymers, with a high glass transition temperature close to 145–150 °C. PC remains nearly amorphous in most of the processing conditions, and displays high mechanical properties (tensile strength, ductility and impact strength), high HDT, high dielectric strength, high transparency and high durability. PC is consequently suitable for numerous high-performances applications the for transportation, electronic and building industries. PLA/PC blends have subsequently attracted significant attention to achieve high-performance and durable applications. These (partly) biobased and cost-effective PLA/PC blends can possibly replace high-impact strength PC/ABS blends. Numerous studies have been dedicated to the processing, compatibilization and (thermo)mechanical properties of PLA/PC blends. Relevant studies on this topic are reviewed here, with a particular focus on compatibilization strategies. The main achievements in terms of morphologies and (thermo)mechanical properties could be found in Table 2.

**Figure 7:**
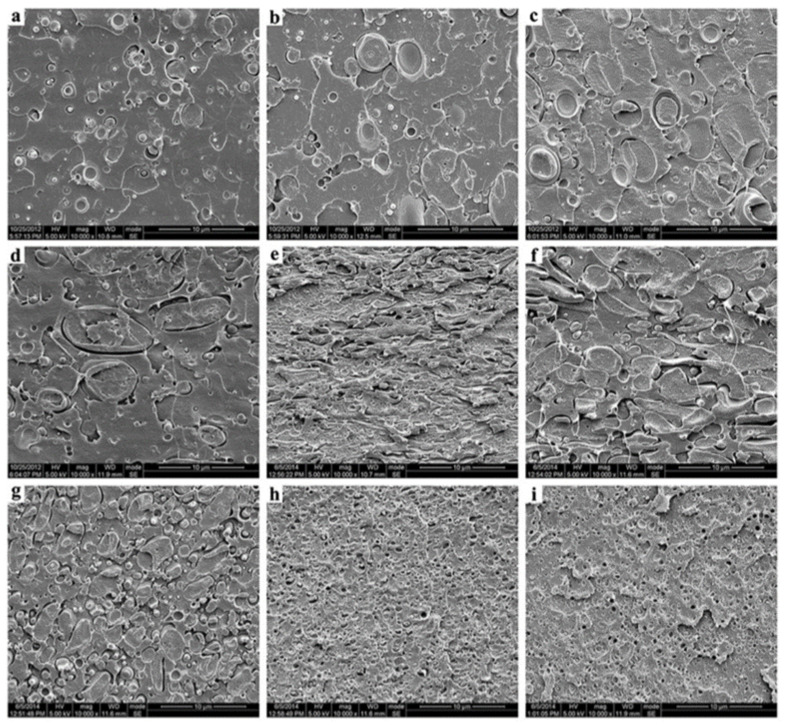
Morphologies of PLA/PC blends with 10, 20, 30, 40, 50, 60, 70, 80 and 90% PC without compatibilizers ((**a**–**i**), respectively, scale bar 10 µm). Reproduced from [90].

PLA/PC could be easily prepared by melt blending in a twin-screw extruder or internal mixer. Melt temperatures in the range 190–230 °C are regularly used, but some studies also processed PLA/PC at higher melt temperatures up to 240–265 °C. PLA/PC blends are immiscible blends with typical matrix/droplet or co-continuous morphologies. Typical PLA/PC morphologies obtained by twin-screw extrusion in the absence of compatibilizers are displayed in Figure 7. Matrix/droplet morphologies are attested for PLA/PC blends containing 10–30% PC and, from SEM analysis, the onset of PC continuity seems to lie close to 40% PC, but no detailed information regarding this important parameter could be found. Co-continuous morphologies are attested for 50–60% PC and up to almost 70% PC. In the absence of compatibilizers, large PC droplets are obtained, with characteristic sizes in the range of 2–5 µm, and large continuous PC domains are also concluded, with characteristic sizes in the range 5–20 µm depending on processing conditions. More importantly, a poor interfacial adhesion with a classical interfacial debonding is systematically concluded by all authors in the absence of compatibilizer. These phenomena (poor PC dispersion into PLA, onset of PC continuity and poor PLA/PC compatibility) are consistent with (i) unfavorable PC/PLA viscosity ratio and (ii) high interfacial tension evaluated to 3.3 mN/m in the melt state [89]. For PLA/PC blends displaying matrix/droplet morphologies (i.e., PC content lower than 30%), poor mechanical properties are observed, with low elongation at break (<20%) and low impact strength (<10 kJ/m^2^). A maximal HDT of 80 °C is attested after thermal annealing. However, co-continuous PLA/PC blends containing 50% PC displayed interesting (thermo)mechanical properties, with a maximal HDT up to 130 °C (after thermal annealing) and maximal elongation at break close to 70%. In this context, despite a poor PLA/PC compatibility, the development of co-continuous PLA/PC blends could be of technological interest, even in the absence of compatibilizer, but the impact strength of these blends requires a careful attention.

Various compatibilizers were used to improve PLA/PC compatibility, with significant morphological and (thermo)mechanical improvements expected. Conventional compatibilizers for PLA/PC blends are reactive copolymers, based either on epoxy or anhydride maleic groups. Interfacial coupling reactions between PLA and PC are expected by specific and fast reactions between PLA/PC end groups and epoxy/anhydride maleic groups. These compatibilizers are used directly during the blending stage by twin-screw extrusion, and various levels of efficiency are attested by several authors.

**Figure 8:**
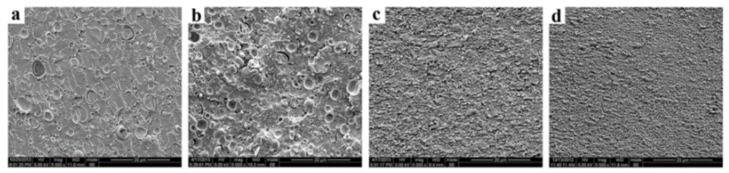
Morphologies of PLA/PC blends with 30% PC compatibilized with ADR (ADR concentration 0, 0.1, 0.3 and 0.5 phr, respectively from (**a**–**d**), scale bar 20 µm). Reproduced from [90].

**Figure 9:**
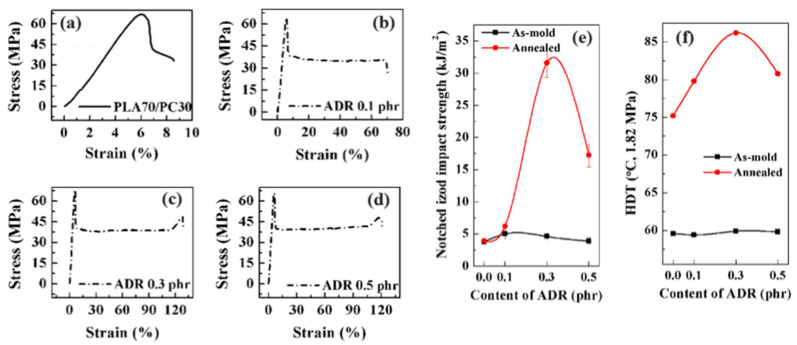
Stress–strain curves (**a**–**d**), impact strength (**e**) and HDT (**f**) for PLA/PC blends containing 30% PC compatibilized with ADR. Adapted from [90].

**Figure 10:**
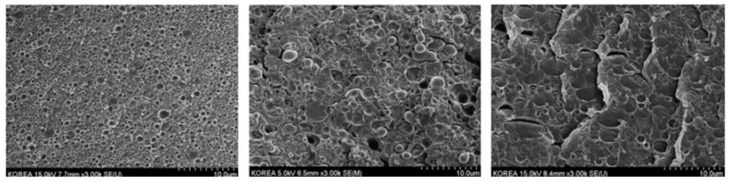
Morphologies of PLA/PC blends containing 30% PC compatibilized with 5 phr of SAN-MAH (**left**), EO-MAH (**middle**), and EGMA (**right**) (scale bar 10 µm). Adapted from [89].

Lin L. et al. used reactive copolymers based on epoxy groups, namely random styrene-glycidyl methacrylate copolymer (ADR) or N,N,N’,N’-tetraglycidyl-4,4′-diaminodiphenyl methane (TGDDM) for the compatibilization of PLA/PC blends containing 30% PC [90]. The best compatibilization efficiency is achieved using ADR, with an optimum concentration of 0.3 phr, and PC droplet sizes lower than 1 µm could be reached (Figure 8). An improved interfacial adhesion is attested for these compatibilized PLA/PC blends. The ductility, the impact strength and the HDT of PLA/PC blends were improved using ADR (elongation at break up to 120%, impact strength up to 30 kJ/m^2^ and HDT up to 85 °C after annealing) (Figure 9). Yemisci F. et al. performed a similar study using reactive copolymers based on maleic anhydride or epoxy groups, namely styrene-acrylic multi-functional-epoxide oligomer (SAMFE) or styrene maleic anhydride copolymer (SMA), for the compatibilization of PLA/PC blends containing 30% PC [91]. The best compatibilization efficiency is observed with the use of SAMFE at a concentration of 1%, and PC droplet sizes down to 1 µm are observed. An improved interfacial adhesion is also attested for these compatibilized PLA/PC blends. The mechanical properties were evaluated, with tensile strength in the range 60–65 MPa but poor elongation at break, close to 10%, being reported for these compatibilized PLA/PC blends. These conflicting data remain unexplained, but could be attributed to the lower compatibilization efficiency of SAMFE and SMA compared to ADR or TGDDM. Lee J. B. et al. also used reactive copolymers based on maleic anhydride or epoxy groups, namely poly(styrene-*g*-acrylonitrile)-maleic anhydride (SAN-MAH), poly(ethylene-*co*-octene) rubber-maleic anhydride (EOR-MAH) or poly(ethylene-*co*-glycidyl methacrylate) (EGMA), for the compatibilization of PLA/PC blends containing 30% PC [89]. SAN-MAH at an optimum concentration of 5 phr was found the most efficient compatibilizer, with PC droplet size down to 0.2 µm representing the lowest value reported from all studies (Figure 10). An improved interfacial adhesion is also attested for these compatibilized PLA/PC blends. Interestingly, the interfacial tension was evaluated to 0.1 mN/m, confirming the high compatibilization efficiency of SAN-MAH. This high compatibilization efficiency could be ascribed to (i) the high processing temperature used in this study, with extrusion temperature up to 265 °C and (ii) the specific affinity of SAN-MAH for the PLA/PC interfaces. Mechanical properties were also evaluated, with tensile strength up to 64 MPa and impact strength close to 400 J/m for PLA/PC blends compatibilized with SAN-MAH (reference value for neat PC close to 700 J/m). From these studies conducted on PLA/PC blends displaying matrix/droplet morphologies, it can be concluded that reactive copolymers based on epoxy or anhydride maleic groups are efficient compatibilizers that decrease PC droplet size and improve interfacial adhesion. High efficiencies are observed for specific copolymers (in particular SAN-MAH) that could be linked to specific affinities for PLA/PC interfaces and/or peculiar extrusion conditions. Significant improvements are achieved in terms of tensile strength, ductility and impact strength, but a maximal HDT of 85 °C after annealing is recorded for these compatibilized PLA/PC blends.

**Figure 11:**
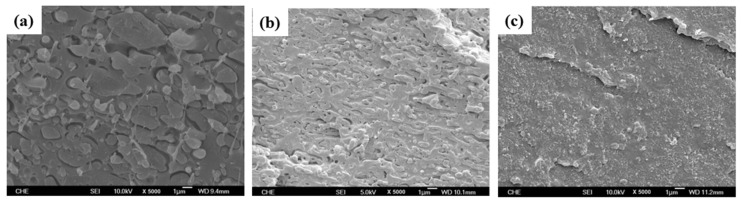
Morphologies of PLA/PC blends containing 50% PC. Non-compatibilized blend (**a**), compatibilized with 10 phr of EP (**b**) and compatibilized with 10 phr EP coupled to 1 phr TBAB (**c**) (scale bar 1 µm). Adapted from [92].

Wang Y. used a reactive copolymer based on epoxy groups, namely epoxy-based additive (EP), combined to tetrabutylammonium bromide (TBAB) for the compatibilization of PLA/PC blends containing 50% PC [92]. Co-continuous morphologies are attested, and the use of EP (10 phr) coupled to TBAB (1 phr) efficiency reduces the size of continuous PC domains down to 500 nm with a high homogeneity (Figure 11). Interfacial adhesion is also largely improved, and authors claim that TBAB significantly improves the rate of coupling reactions between EP and PLA/PC. Interestingly, the HDT of the compatibilized PLA/PC reaches nearly 130 °C without annealing, compared to 80 °C for non-compatibilized blends. However, the major drawback lies in the poor impact strength recorded for these compatibilized and co-continuous PLA/PC blends. Yuryev Y. et al. used a reactive copolymer based on epoxy groups, namely poly(ethylene-n-butylene acrylate-glycidyl methacrylate) (EBA-GMA), for the compatibilization of PLA/PC blends containing 60% PC [93]. Co-continuous morphologies are also achieved. AFM studies specifically attest that EBA-GMA is located at the PLA/PC interface, and improves interfacial adhesion. Competitive (thermo)mechanical properties are reported (tensile strength 57 MPa, elongation at break 67%, impact strength 715 J/m and HDT 134 °C without annealing) with values close to PC/ABS blends. These co-continuous PLA/PC blends, compatibilized by EBA-GMA at a concentration of 6%, clearly represent one of most relevant formulations for high-performance and durable applications.

Actually, non-compatibilized and co-continuous PLA/PC blends could also display interesting (thermo)mechanical properties (tensile strength, ductility and HDT), except for impact strength, which remains quite poor (approx. 10–40 J/m vs. 700 J/m for neat PC). From a morphological point of view, EP/TBAB are efficient compatibilizers, but fail to improve the impact strength (approx. 10 J/m). To the contrary, EBA-GMA displays a moderate compatibilization efficiency, but a dramatic improvement of the impact strength up to 715 J/m is observed for co-continuous PLA/PC blends incorporating EBA-GMA. In this context, such elastomeric and reactive additives could play a dual role as compatibilizer and impact modifier located at the PLA/PC interface, due to the fact that coupling reactions and conventional compatibilizers are moderately relevant for co-continuous PLA/PC blends. In this respect, significant attention has been consequently paid to the use of various elastomeric additives coupled to conventional compatibilizers during the twin-screw extrusion processing of PLA/PC blends incorporating at least 50% PC. Wang Y. reported about the use of poly(butylene succinate-*co*-L-Lactate) (PBSL) in compatibilized PLA/PC blends containing 50% PC [92]. The incorporation of 10% PBSL coupled to EP/TBAB yields remarkable (thermo)mechanical performances, with a HDT as high as 95 °C and an impact strength close to 34 J/m. The co-continuous morphology of PLA/PC blends seems to be highly modified with the use of PBSL that mainly locates in the PC phase, and non-continuous PC/PBSL domains with high characteristic sizes (>20 µm) are generated. Hashima K et al. similarly reported about the use of hydrogenated styrene-butadiene-styrene block copolymers (SEBS) in compatibilized PLA/PC blends containing 50% PC [94]. Poly(ethylene-*co*-glycidyl methacrylate) (EGMA), at a concentration of 5 phr, is also added as a conventional compatibilizer. The incorporation of 15% SEBS coupled to EGMA similarly yields remarkable (thermo)mechanical properties, with a HDT as high as 95 °C and an impact strength close to 60 kJ/m^2^ (value close to neat PC). The co-continuous morphology of PLA/PC is here unaffected, with PC domain sizes down to 1 µm, and SEBS seems to be mainly dispersed into the PLA phase. Such ternary morphology could explain the high (thermo)mechanical properties of these complex PLA/PC blends. In conclusion, co-continuous PLA/PC blends are of technological interest for high-performance and durable applications. Reactive compatibilizers based on epoxy groups could help in reaching high HDT values up to 130 °C, but elastomeric additives are mandatory to reach outstanding impact strength. Their location needs to be carefully controlled to obtain a full balance between tensile/impact strength, ductility and HDT, but these complex formulations systematically display intermediate HDT values close to 100 °C.

**Figure 12:**
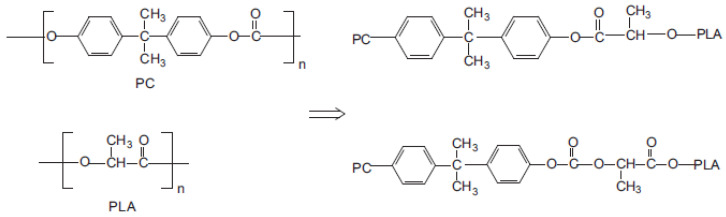
Proposed mechanism for the compatibilization of PLA/PC using specific catalysts active for ester-carbonate exchange reactions. Reproduced from [95].

Another strategy reported for the compatibilization of PLA/PC blends is based on the specific catalysts active for ester-carbonate exchange reactions. Main species include well-known catalysts for transesterification reactions. The advantage of this approach lies in the potential in situ production of PLA-*co*-PC copolymers during twin-screw extrusion processing, according to the reaction scheme displayed in Figure 12. Conventional compatibilizers are here eliminated from the formulations that theoretically make this approach more economically relevant. Lui C. et al. used zinc borate (BSX), titanium pigment (TiO_2_), tetrabutyl titanate (TT-01) or antimony trioxide (Sb_2_O_3_) for the compatibilization of PLA/PC blends containing 30% PC [95]. TT-01, at a concentration of 0.5%, was found to be the most efficient catalyst, with a reduction in the PC droplet size down to 2 µm. PLA-*co*-PC copolymers are detected and quantified by various techniques, including nuclear magnetic resonances (NMR) and size exclusion chromatography (SEC). A third phase attributed to these copolymers is also attested by dynamic mechanical analysis (DMA), with an intermediate relaxation temperature between neat PLA and neat PC. Tensile/impact strength, ductility and HDT are not reported, but an improvement of the HDT could be expected from this study. Phuong V. T. et al. used triacetin (TA) and tetrabutylammonium tetraphenylborate (TBATPB) for the compatibilization of PLA/PC containing up to 60% PC [96]. Uncommon trends are revealed in terms of blend morphology. For PC content lower than 30%, the size of the PC droplet increases to approx. 5 µm with the use of TA coupled to TBATPB, at concentrations of 5% and 0.2%, respectively. The strong modification of PC continuity onset is noticed, and a full continuity is observed at 40% PC with large PC domains sizes. However, the authors draw attention to the significant compatibilization efficiency of TA coupled to TBATPB with an improved interfacial adhesion, and effective production of PLA-*co*-PC copolymers detected by NMR, SEC and DMA. The authors attribute these morphological effects to PLA plasticization and degradation with strong modifications of the viscosity ratio. A tensile strength between 50–60 MPa and an elongation at break up to 120% are reported, but the effect of the TA/TBATPB on the final (thermo)mechanical properties appears quite low. The use of the impact modifier also seems to be mandatory, and Zhou Y. at al. reported about the use of thermoplastic polyurethane (TPU) in compatibilized PLA/PC containing up to 50–70% PC [97]. Di-n-butyltin oxide (DBTO) is added to promote ester-carbonate exchange reactions. PLA droplets with a slight PLA continuity is expected, but morphological analysis reveals complex morphologies without clear conclusions about PLA droplet sizes and the location of TPU. Authors reported about the tensile strength between 35 and 45 MPa, an elongation at break between 5 and 15% and an impact strength between 35 and 50 kJ/m^2^, but the effect of TPU/DBTO on the final (thermo)mechanical properties also appears quite low. In conclusion, the compatibilization of PLA/PC blends, in the production of only specific catalysts, is feasible during twin-screw extrusion processing but, compared to conventional compatibilizers, a moderate compatibilization efficiency is concluded for this approach with peculiar morphological effects. The final (thermo)mechanical properties are currently not satisfactory for high-performance and durable applications, but the generation of a third phase linked to the in situ production of PLA-*co*-PC copolymers clearly represent an interesting phenomenon that is of interest for further studies, in particular regarding a facile manufacturing of cost-effective PLA/PC blends with co-continuous morphologies and enhanced HDT.

As a general conclusion, immiscible PLA/PC blends could represent potential candidates for high-performance and durable applications. From a morphological point of view, a compatibilization of these blends is mandatory, to reduce the characteristics of the PC droplet or domain size down to 1 µm (even more for some compatibilizers in peculiar processing conditions) and improve interfacial adhesion. Several reactive copolymers based either on epoxy or anhydride maleic groups are particularly efficient. In terms of the final (thermo)mechanical properties, co-continuous PLA/PC are of interest to reach a HDT higher than 100 °C and a high impact strength close to PC values. However, a careful selection/combination of compatibilizers/impact modifiers is required. The use of specific catalysts could also represent a valuable option, in particular regarding the in situ production of PLA-*co*-PC copolymers during twin-screw extrusion processing, but in-depth investigations are required in this field.

**Table 2:** Main achievements and relevant information extracted from various studies dedicated to non-compatibilized and compatibilized PLA/PC blends.

Compatibilizers	Achievements and Relevant Information	Ref.
None	Blends prepared by internal mixer at 190 °C Matrix/droplet morphologies for 10–30% PC into PLA with droplet size between 5 and 10 µm–poor interfacial adhesion; co-continuity observed for 50% PC with domain size > 10 µm–tensile strength 40–60 MPa, ductility 3–16%.	[98]
Random styrene-glycidyl methacrylate copolymer (ADR) *N*,*N*,*N*’,*N*’-tetraglycidyl-4,4′-diaminodiphenyl methane (TGDDM)	Blends prepared by twin-screw extrusion at 190–230 °C Without compatibilizer—matrix/droplet morphologies for 10–30% PC into PLA, with droplet size between 2 and 5 µm—poor interfacial adhesion, tensile strength 60–65 MPa, ductility 5–10%, impact strength 5–10 kJ/m^2^, HDT 65–80 °C (after annealing). Without compatibilizer—co-continuity observed for 50% PC into PLA with domain size > 5 µm—tensile strength up to 65 MPa, ductility 70%, impact strength 12 kJ/m^2^, HDT up to 130 °C (after annealing). Best compatibilization efficiency of ADR (0.3 phr) with domain size down to 1 µm—strong morphological modifications observed with PC continuity at 30% PC—tensile strength 65 MPa, ductility 100%, impact strength 30 kJ/m^2^, HDT 85 °C (after annealing).	[90]
Styrene-acrylic multi-functional-epoxide oligomer (SAMFE, (Joncryl^®^) Styrene maleic anhydride copolymer (SMA, Joncryl^®^)	Blends prepared by twin-screw extrusion at 200 °C Without compatibilizer—matrix/droplet morphologies for 10–30% PC into PLA with droplet size close to 2 µm—tensile strength 55–60 MPa and ductility 30–70%. Best compatibilization efficiency of SAMFE (1%) with droplet size down to 1 µm—tensile strength 60–65 MPa and ductility 10%.	[89]
Poly(styrene-*g*-acrylonitrile)-maleic anhydride (SAN-MAH) Poly(ethylene-*co*-octene) rubber-maleic anhydride (EOR-MAH) Poly(ethylene-*co*-glycidyl methacrylate) (EGMA)	Blends prepared by twin-screw extrusion at 240–260 °C Without compatibilizer—matrix/droplet morphologies for 30% PC into PLA with droplet size close to 2 µm—poor interfacial adhesion, tensile strength 55 MPa and impact strength 230 J/m. High compatibilization efficiency of SAN-MAH (5 phr) with droplet size down to 200 nm—reduced interfacial tension from 3.3 mN m^−1^ down to 0.1 mN/m, tensile strength 64 MPa and impact strength 400 J/m.	[89]
Epoxy-based additive (EP) Tetrabutylammonium bromide (TBAB)	Blends prepared by twin-screw extrusion at 220 °C Without compatibilizer—co-continuity observed at 50% PC into PLA with domain size > 10 µm—poor interfacial adhesion, impact strength 10 J/m and HDT 80 °C. High compatibilization efficiency of EP (10 phr) combined to TBAB (1 phr) with homogenous domain size down to 500 nm—impact strength 7 J/m and HDT up to 130 °C—improved toughness using PBSL but a reduction in HDT.	[92]
Poly(ethylene-n-butylene acrylate-glycidyl methacrylate) (EBA-GMA)	Blends prepared by twin-screw extrusion at 265 °C Without compatibilizer—PC-rich blends with 40% PLA—tensile strength 68 MPa, ductility 73%, impact strength 45 J/m and HDT 135 °C (after annealing). No compatibilization evidence for EBA-GMA (6%)—tensile strength 57 MPa, ductility 67%, impact strength 715 J/m and HDT 133 °C—similar value to PC/ABS blends.	[93]
Poly(ethylene-*co*-glycidyl methacrylate) (EGMA)	Blends prepared by twin-screw extrusion at 200 °C PLA/PC/SEBS/EGMA blends with complex morphologies—PC droplet or domain size < 1 µm—ductility 50–120%, impact strength up to 65 kJ/m^2^, HDT up to 95 °C—significant compatibilization efficiency of EGMA (5 phr) and improved impact strength using SEBS.	[94]
Zinc borate (BSX) Titanium pigment (TiO_2_) Tetrabutyl titanate (TT-01) Antimony trioxide (Sb_2_O_3_)	Blends prepared by internal mixer at 230 °C Without compatibilizer—matrix/droplet morphologies for 30% PC into PLA with heterogenous droplet size 5–20 µm—transesterification reactions observed at elevated mixing time. Best compatibilization efficiency of TT-01 with droplet size down to 2–5 µm—appearance of third phase attributed to PLA-PC copolymers formed at elevated mixing time.	[95]
Triacetin (TA) Tetrabutylammonium tetraphenylborate (TBATPB)	Blends prepared by twin-screw extrusion at 210–230 °C Without compatibilizer—matrix/droplet morphologies observed for 20—40% PC into PLA with droplet size 2—10 µm—poor interfacial adhesion, tensile strength 52–55 MPa, ductility 2–3%. Without compatibilizer—co-continuous morphologies observed at 60% PC into PLA—tensile strength 55 MPa, ductility up to 125%. Significant compatibilization efficiency of TA (5%) coupled to TBATPB (0.2%)—Strong morphological modifications observed with PC continuity at 40% PC—appearance of third phase attributed to PLA-PC copolymers—tensile strength 58–60 MPa, ductility 125%.	[96]
Di-n-butyltin oxide (DBTO)	Blends prepared by twin-screw extrusion at 210–230 °C PC/PLA/TPU/DBTO blends with complex morphologies—PLA droplet or domain size between 2 and 5 µm—tensile strength 35–50 MPa, ductility 16%, impact strength 48 kJ/m^2^—slight compatibilization efficiency of DBTO (1%) and improved toughness using TPU.	[97]

### 3.3. PLA-Based Blends with Poly(Ethylene Terephthalate) (PET) or Poly(Butylene Terephthalate) (PBT)

Polyethylene terephthalate (PET), polybutylene terephthalate (PBT) and polytrimethylene terephthalate (PTT) are important semi-crystalline engineering thermoplastics that are suitable for blending with PLA, owing to their high thermal resistance, excellent mechanical properties, good dimensional stability and chemical resistance [3,25,99,100,101,102,103,104,105]. One main limitation lies in their relatively high melting temperatures, up to 260 °C for PET. The processing of PLA/PET blends or PLA/PBT blends, consequently, requires high extrusion temperatures that potentially produce an intensive degradation of PLA. However, several studies have been dedicated to the processing, compatibilization and (thermo)mechanical properties of PLA/PET and PLA/PBT blends. Most of the studies used PET or PBT as matrices, with the incorporation of PLA as the minor phase, i.e., PET-rich or PBT-rich blends. Relevant studies on this topic are reviewed here, with a particular focus on compatibilization strategies. The main achievements, in terms of morphologies and (thermo)mechanical properties, can be found in Table 3 and Table 4.

PLA/PET blends were prepared by single-screw, twin-screw extruders or internal mixers, at processing temperatures close to 270 °C and short residence times (typically 3–8 min). Immiscible PLA/PET blends are attested with a majority of droplet/matrix morphologies. Torres-Huerta et al. observed that the incorporation of small amounts of PLA (typically 1%) into PET yields transparent materials with ultra-small PLA droplets [104]. PLA droplet diameters increases to approx. 2–5 µm for PLA concentrations between 7.5 and 15% into PET. These high droplet sizes attest for a strongly unfavorable viscosity ratio between PET and PLA at such elevated processing temperatures. In terms of mechanical properties, the optimum loading was found to be 2.5% PLA into PET, with tensile strengths up to 68 MPa and ductility up to 80%. PLA concentrations higher than 7.5% into PET drastically reduced the tensile strength and impact strength of the blend. Similar conclusions were given by McLauchlin et al. [106] and by La Mantia et al. [102], with low tensile strengths down to 34–52 MPa for 10–20% PLA into PET. However, based on current micrographs, most of the authors point out that the interfacial adhesion between PLA and PET is quite good. Spontaneous ester–ester exchange reactions are plausible at elevated temperatures, probably mediated by the residual catalysts in PET active for transesterification reactions between PLA and PET. This could result in the in situ production of PET-*co*-PLA copolymers during twin-screw extrusion processing, serving as compatibilizers. These reactions could be favored by PLA degradation at elevated processing temperatures in the presence of PET.

**Figure 13:**
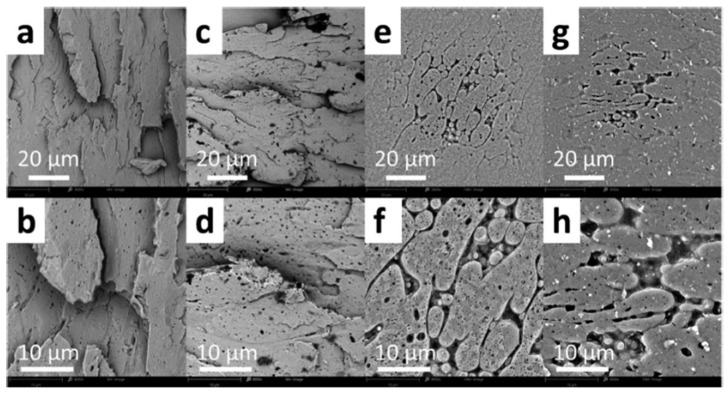
Morphologies of PET/PLA blends with 30% PLA produced by twin-screw extrusion in the presence of various amounts of SA-GMA additives. 0.3% SA-GMA (**a**,**b**), 0.5% SA-GMA (**c**,**d**), 0.7% SA-GMA (**e**,**f**) and 1% SMA-GMA (**g**,**h**). Reproduced from [107].

In order to limit PLA degradation while maintaining efficient compatibilization with PET, You X. et al. used multifunctional epoxy additives such as poly(ethylene-n-butylene-acrylate-*co*-glycidyl methacrylate) (EBA-GMA) or poly(styrene-acrylic-*co*-glycidyl methacrylate) (SA-GMA) into PET-rich blends with up to 30% PLA [107]. These additives can serve as compatibilizers, but also as impact modifiers and rheological stabilizers. The best compatibilization efficiency is observed with SA-GMA at a concentration of 0.7%. Initial matrix/droplet morphologies turn to complex morphologies, in particular refined co-continuous structures with PLA domain sizes down to approx. 1 µm (Figure 13). This additive drastically improved the mechanical properties of PET/PLA blends reaching a tensile strength up to 70 MPa, ductility up to 120% and a moderate impact strength close to 30 J/m. These values are close to the initial values of PET. Thermal stabilities were reported in the range 60–70 °C for compatibilized blends. More importantly, SA-GMA not only act as a classical compatibilizer but also maintain acceptable rheological properties for PET/PLA blends, by favoring intensive coupling reactions in the melt state during extrusion processing. The formation of partially crosslinked architectures is suspected, in accordance with various studies dealing with the processing of polyesters in the presence such additives [108,109,110]. This study consequently demonstrates that, despite large differences between PET and PLA in terms of melting temperatures (with high risks of PLA degradation) and viscosity ratios, these blends can be easily processed by twin-screw extrusion in the presence of suitable reactive compatibilizers (in particular epoxy-based additives) that could provide PET/PLA blends with noticeable (thermo)mechanical properties.

**Figure 14:**
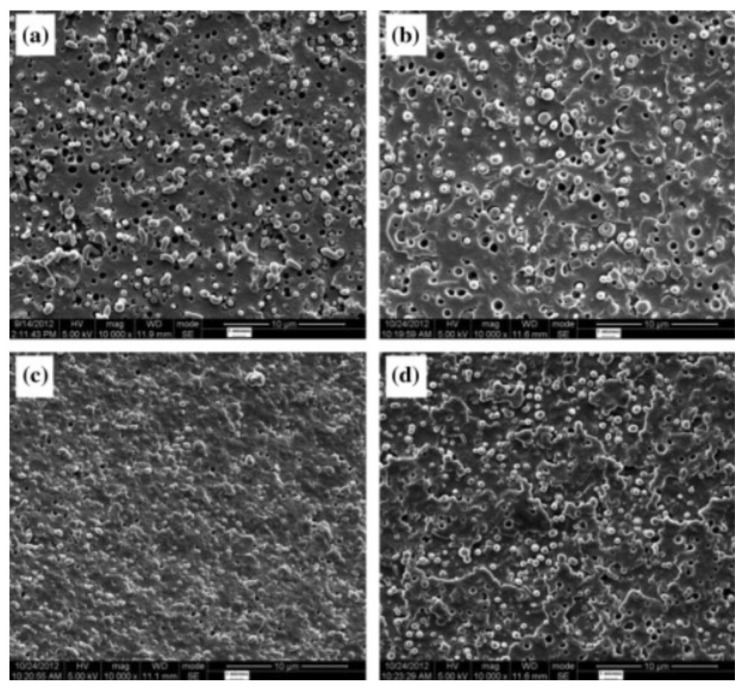
Morphologies of PLA/PETG blends with 20% PETG produced by twin-screw extrusion in the presence of various amounts of PLA-*g*-MA. No compatibilizer (**a**), 1% PLA-*g*-MA (**b**), 3% PLA-*g*-MA (**c**) and 5% PLA-*g*-MA (**d**). Reproduced from [111].

Only limited information is reported about PLA-rich blends with PET, but an interesting study was reported by Jiang et al. about PLA-rich blends with 20% of amorphous PET (PETG) [111]. This PET analogue displays lower processing temperatures, and PLA/PETG could represent a valuable alternative to PLA/PET blends. PLA-*graft*-maleic anhydride (PLA-*g*-MA) was used as compatibilizer, and classical matrix/droplet morphologies were achieved (Figure 14). Using rheological data coupled to emulsion models such as the Palierne model, the authors showed that PLA-*g*-MA is a very efficient compatibilizer that reduces (i) the interfacial tension between PLA and PETG from 2 mN/m down to 0.85 mN/m and (ii) the droplet size of PETG from 800 nm down to 500 nm (Figure 14). The interfacial adhesion is also slightly improved between PLA and PETG by the use of 5% PLA-*g*-MA. Significant enhancements in terms of tensile strength and ductility were observed upon the incorporation of the compatibilizer. Compatibilized PLA/PETG blends display tensile strengths up to 83 MPa, with ductility close to 30%. However, the impact strengths and thermal stabilities were not reported. These results indicated an interesting potential of such compatibilized PLA/PETG blends. These blends could be a valuable alternative to PLA/PET, with a reduced PLA degradation and favorable viscosity ratio. Deeper studies on their thermomechanical properties are required for PLA/PETG blends.

**Table 3:** Main achievements and relevant information extracted from various studies dedicated to non-compatibilized and compatibilized PLA/PET blends.

Compatibilizers	Achievements and Relevant Information	Ref.
None	Blends prepared by single screw extrusion at 240–250 °C Matrix/droplet morphologies for 7.5% PLA into PET with droplet size close to 5 µm—good interfacial adhesion—tensile strength up to 68 MPa, ductility up to 80%, impact strength 10 J/m.	[104]
None	Blends prepared by injection molding at 265 °C Matrix/droplet morphologies for 20% PLA into PET with droplet size up to 5 µm—good interfacial adhesion—tensile strength 52 MPa, ductility between 50 and 200%, impact strength 35 kJ/m^2^.	[106]
None	Blends prepared by single-screw extrusion at 260 °C Matrix/droplet morphologies for 5–15% PLA into PET with droplet size between 0.5 and 1.9 µm–Good interfacial adhesion.	[103]
None	Blends prepared by internal mixer at 270 °C. Matrix/droplet morphologies for 1–5% PLA into PET with droplet size lower than 1 µm–good interfacial adhesion–tensile strength 34 MPa, ductility 400%.	[102]
Poly(ethylene-n-butylene-acrylate-*co*-glycidyl methacrylate) (EBA-GMA) Poly(styrene-acrylic-*co*-glycidyl methacrylate) (SA-GMA)	Blends prepared by twin-screw extrusion at 270 °C Without compatibilizers–matrix/droplet morphologies up to 30% PLA into PET with droplet size between 1.5 and 3.6 µm–good interfacial adhesion, tensile strength 71 MPa, ductility 5%, impact strength 7 J/m, HDT 62.3 °C. Best compatibilization efficiency of SA-GMA (0.7%)–co-continuous morphologies for 30% PLA into PET with domain size between 1 and 5 µm—tensile strength 70 MPa, ductility 110%, impact strength 30 J/m, HDT 68.3 °C.	[107]
Poly(Lactide-*g*-maleic anhydride) (PLA-*g*-MA)	Blends prepared by internal mixer at 190 °C Without compatibilizers—matrix/droplet morphologies up to 20% PETG into PLA with droplet size down to 0.48 µm—poor interfacial adhesion, interfacial tension 2 mN m^−1^, tensile strength 81 MPa, elongation at break 7%. Significant compatibilization efficiency of PLA-*g*-MA (5%)—matrix/droplet morphologies for 20% PETG with droplet size down to 230 nm—reduced interfacial tension from 2 mN m^−1^ down to 0.85 mN m^−1^, tensile strength 83 MPa, elongation at break 30%.	[111]

**Figure 15:**
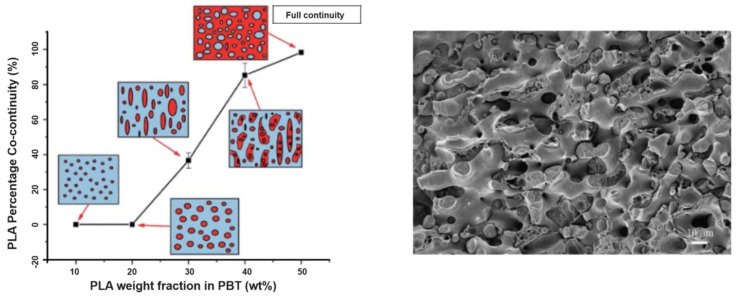
Morphology evolution in PBT/PLA blends without compatibilizers as a function of PLA content (**left**). Reproduced from [114]. Co-continuous morphologies with multi-level structures observed for PLA/PBT with 50% PBT (**right**). Reproduced from [118].

PLA/PBT blends represents another interesting association that could be prepared by twin-screw extrusion and internal mixers with processing temperatures close to 220–250 °C. These processing temperatures are slightly lower than the ones required for the processing of PLA/PET blends and could reduce the degradation of PLA during the blending process. Non-compatibilized PLA/PBT blends are mentioned in several studies [112]. Matrix/droplet morphologies are attested up to 30% PBT, with PBT droplet sizes up to 5 µm. The viscosity ratios between PLA and PBT also seem to be quite unfavorable for producing refined matrix/droplet morphologies. The interfacial adhesion between PLA and PBT is poorly discussed but, from microstructural analysis, a moderate adhesion level can be concluded. These blends could exhibit interesting tensile strengths close to 60–65 MPa, but low ductility (<10%) and impact strength (2 kJ/m^2^) are reported [113]. PBT-rich blends with PLA were deeply investigated by Chang B. et al. [114]. PBT/PLA blends show matrix/droplet morphologies up to 20% PLA in PBT, and PLA droplet sizes were also found to be between 1 and 5 µm (Figure 15). Based on current micrographs, the interfacial adhesion between PLA and PBT seems to be relatively poor by comparison with PET/PLA blends. A lower amount of spontaneous transesterification reactions between PLA and PBT could be expected during the twin-screw extrusion processing at 220–250 °C. Interesting fibrillar morphologies are reported with 30% of PLA in PBT, and these phenomena could arise from rheological considerations, with a suitable viscosity/elasticity ratio between PBT and PLA phases [53,115,116,117]. However, all these PBT/PLA formulations display low mechanical properties, with tensile strength in range 50–55 MPa, ductility lower than 10% and impact strength close to 30 J/m [114]. Co-continuous morphologies appear between 40% and 60% PBT in PLA and, interestingly, these formulations give rise to peculiar multi-level structures, i.e., co-continuous morphologies with domain sizes in the range 5–15 µm incorporating submicronic inclusions into continuous PLA or PBT phases (Figure 14). These structures are attested by several authors [114,118], and little explanation is given about their formation during extrusion processing. Rheological considerations, combined with peculiar processing conditions, are plausible along with spontaneous formation of a small amount of PLA-*co*-PBT copolymers by ester–ester exchange reactions in the melt state. In terms of mechanical properties, contradictory results are observed for these co-continuous PLA/PBT blends, but a high ductility of 160% is reported for PLA/PBT blends with 40% PBT. Impact strengths and heat resistances are not deeply investigated for this particular formulation processed without compatibilizer. Future investigations could be of interest because, in addition to interesting multi-level morphologies, peculiar crystallization behaviors are also reported, with enhanced PLA crystallization rate due to the presence of PBT phase [118]. PBT displays a high crystallinity and a crystallization rate that could favor PLA crystallization from PBT interfaces.

Various compatibilizers were attempted for PLA/PBT blends, such as ethylene butyl acrylate copolymer grafted with maleic anhydride (EBA-*g*-MAH) [113], para-phenylene diisocyanate (PPDI) [118], ethylene glycidyl methacrylate copolymers [119] and epoxy-functionalized styrene-acrylate copolymer (ESAC) [114]. Compatibilization reactions classically consist of mutual reactions with hydroxyl or carboxylic acid end groups of PBT and PLA via copolymers bearing epoxy and anhydride moieties. EBA-*g*-MAH was used in PLA/PBT blends, incorporating up to 15% PBT [119]. No significant improvements are reported in terms of morphologies and mechanical properties. These PLA/PBT blends remain highly brittle close to PLA values, and copolymers with anhydride moieties are poorly efficient for the compatibilization of PLA/PBT blends. The use of PPDI, at a concentration of 1%, into a PLA/PBT blends containing 50% PBT was attempted by Kim M. W. et al. [118]. Co-continuous morphologies with multi-level structures are maintained using PPDI and a moderate morphological refinement is attested. The domain size of continuous PLA/PBT phases reduces from 10–15 µm down to 5–10 µm. The compatibilization efficiency of PPDI for PLA/PBT blends is consequently significant. This study also sheds light on various features, in particular the role of the specific coupling reactions mediated by PPDI for the formation of multi-level structures into co-continuous PLA/PBT blends. The role of PBT interfaces on PLA crystallization is also confirmed, because PPDI enhanced this phenomenon of particular interest to reduce the cycle time in the injection molding of PLA/PBT blends.

**Figure 16:**
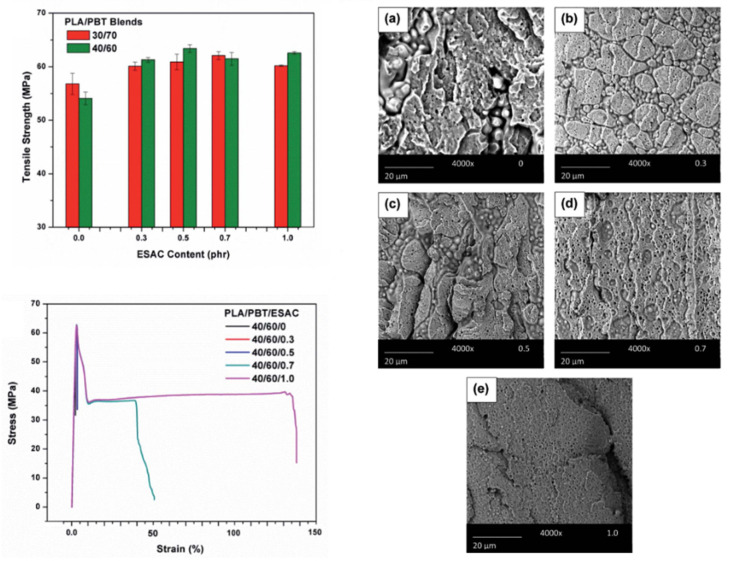
Mechanical properties of PBT-rich blends with 40% PLA and ESAC as compatibilizer with corresponding morphologies (ESAC concentration 0%, 0.3%, 0.5%, 0.7% and 1%, (**a**–**e**), respectively). Reproduced/adapted from [114].

A high compatibilization efficiency is reported for epoxy-functionalized styrene-acrylate copolymer (ESAC) [114]. In the presence of 1% ESAC into PBT-rich blends with 40% PLA, strong modifications of the morphologies are attested (Figure 16). Initial co-continuous morphologies turn into matrix/droplet morphologies with PLA droplets lower than 1 µm. The strong efficiency of ESAC arises from intensive coupling reactions in the melt state, but also from significant modifications of the viscosity ratio between PBT and PLA due to the formation of partially crosslinked structures [108,109,110]. Compatibilized PBT/PLA blends display high tensile strengths close to 60 MPa, with ductility up to 150% (Figure 16). However, the impact strength of these compatibilized blends is maintained close to initial values, and the addition of a second compatibilizer (EBA-GMA) is necessary [114]. Complex morphologies are obtained with improved impact strengths from 30 to 95 J/m and a classical reduction in the tensile strength. Similar efficiencies are reported by Santos et al. for another copolymer bearing epoxy moieties, in particular ethylene glycidyl methacrylate copolymers in PLA-rich blends with 10% PBT [119]. Matrix/droplet morphologies are interestingly refined, down to 200 nm, attesting for the significant compatibilization efficiency of this copolymer. The tensile strength was reduced to 50–55 MPa, but significant improvements are observed on ductility up to 50%. Impact strength also strongly increases up to 200 J/m, with a transition from brittle to ductile behavior for these compatibilized blends, and ethylene glycidyl methacrylate copolymers clearly act as an interesting dual compatibilizer and impact modifier. Epoxy-based copolymers are consequently well-adapted for the compatibilization of PLA/PBT blends, and further studies could be of interest, in particular on various aspects related to the multi-level morphologies, crystallization and thermal resistances of these blends.

In conclusion, immiscible PLA/PET and PLA/PBT blends theoretically display some interesting features for high-performance and durable applications with huge technological potentials. PLA/PET blends are challenging due to high processing temperatures (up to 250–270 °C). PLA/PET blends seem to display an inherent compatibility without any additives, an effect probably linked to spontaneous transesterification reactions. However, current PLA/PET blends are of low interest, with minor improvements in terms of (thermo)mechanical properties. The case of PLA/PBT is probably less challenging, but compatibilization is crucial due to a significant incompatibility between PLA and PBT. Some efficient compatibilizers are noticed, and interesting (thermo)mechanical properties are already achieved for PLA/PBT blends in terms of tensile strength, ductility and thermal resistance. Two unique features are observed for PLA/PBT blends, i.e., co-continuous morphologies with multi-level morphologies and PLA crystallization from PBT. These effects deserve careful attention to reveal the full potential of these PLA/PBT blends. Globally, the scientific literature is scarce on these PLA/PET and PLA/PBT blends, and new in-depth investigations are necessary to take advantage of spontaneous transesterification reactions, manage rheological considerations, and specify morphological features along with final properties.

**Table 4:** Main achievements and relevant information extracted from various studies dedicated to non-compatibilized and compatibilized PLA/PBT blends.

Compatibilizers	Achievements and Relevant Information	Ref.
None	Blends prepared by internal mixer at 250 °C Matrix/droplet morphologies up to 30% of PBT into PLA with droplet size ranging from 1 to 5 µm–fair interfacial adhesion; co-continuous morphologies with multi-level structures for 40% PBT–ductility close to 160%.	[112]
Paraphenylene diisocyanate (PPDI)	Blends prepared by twin screw extrusion at 230–240 °C Without compatibilizers–co-continuous morphologies with multi-level structures for 50% PBT with domain sizes between 10 and 15 µm. Small compatibilization efficiency of PPDI (1%)—co-continuous morphologies with multi-level structures with domain sizes between 5 and 10 µm.	[118]
Ethylene-glycidyl methacrylate copolymer (Lotader AX 8840)	Blends prepared by twin screw extrusion at 220–240 °C Significant compatibilization efficiency of ethylene-glycidyl methacrylate copolymer—matrix/droplet morphology up to 10% of PBT into PLA with droplet size down to 200 nm—tensile strength between 50 and 55 MPa, ductility up to 25%, impact strength 200 J/m.	[119]
Epoxy styrene-acrylate copolymer (ESAC) Poly(ethylene-n-butyl-acrylate-*co*-glycidyl methacrylate) (EBA-GMA)	Blends prepared by twin screw micro-extrusion at 250 °C Without compatibilizers—matrix/droplet morphology up to 20% of PLA into PBT with droplet size approx. 1 µm–poor interfacial adhesion—co-continuous and multi-level structures observed for 40% PLA into PBT—tensile strength 50–55 MPa, ductility < 10%, impact strength 30 J/m. Strong compatibilization efficiency of ESAC (1%)—matrix/droplet morphology up to 40% of PLA into PBT with droplet size approx. 1 µm, tensile strength 60 MPa, ductility 160%, impact strength 30 J/m—combined effects of ESAC and EBA-GMA on impact strength.	[114]
Ethylene butyl acrylate copolymer grafted with maleic anhydride (EBA-*g*-MAH)	Blends prepared by twin screw extruder at 220–230 °C Without compatibilizer—matrix/droplet morphologies up to 15% PBT in PLA–tensile strength 64 MPa, ductility 2%, impact strength 2 kJ/m^2^. Very poor compatibilization efficiency of EBA-*g*-MAH—tensile strength 59 MPa, ductility 3%, impact strength 2.5 kJ/m^2^.	[113]

### 3.4. PLA-Based Blends with (Partly Biobased) Polyamides

Polyamides represent a vast class of engineering polymers, with high thermal resistance, high ductility/toughness, good dimensional stability, good barrier properties and chemical resistance to various solvents. Polyamides commercially exist in different forms by varying the length of the aliphatic part. The main polyamides are PA6, PA6-6, PA6-10, PA10-10, PA11 and PA12. Their properties are given in Table 5 and a large range of engineering properties are available. In particular, the melting points of PA can be modulated between 220 and 180 °C. PA are also available in various injection and extrusion grades, with a large range of melt viscosities. Some biobased PA are also produced at the industrial scale. PA consequently represent interesting partners for blending with PLA, in order to develop high-performance PLA-based materials with balanced and highly tunable properties while maintaining high biobased content. In this context, many studies were recently engaged on PLA/PA6, PLA/PA6-10, PLA/PA10-10, PLA/PA10-12, PLA/PA11 and PLA/PA12 blends with and without compatibilizers. The relevant studies on this topic are reviewed here, with a particular focus on compatibilization strategies. The main achievements, in terms of morphologies and (thermo)mechanical properties, can be found in Table 6.

**Figure 17:**
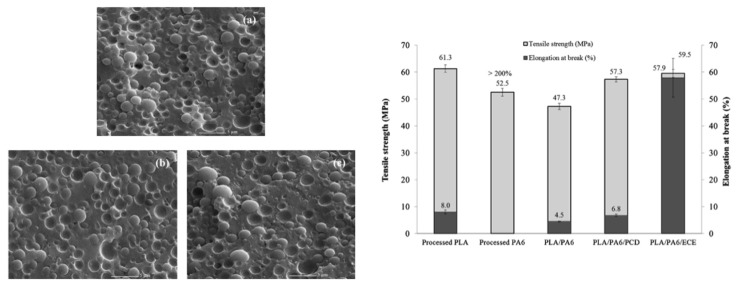
Morphologies of PLA/PA6 blends without compatibilizer (**a**), with PCD (**b**), with ECE (**c**) and their corresponding mechanical properties. Reproduced/adapted from [125].

PLA/PA6 blends could be prepared in internal mixers and twin-screw extruders at temperatures ranging from 220 to 250 °C [124,125,126,127,128]. These temperatures are nearly similar to the processing conditions of PLA/PBT blends. A significant degradation of PLA could be observed with rheological issues. These effects have been investigated in detail by Khankrua R. et al. for PLA-rich blends with PA6 [125]. Non-compatibilized PLA/PA6 blends display matrix/droplet morphologies up to 30% PA6 with PA6 droplet sizes close to 2 µm [125]. A poor interfacial adhesion between PLA and PA6 is attested (Figure 17). However, most of the studies deal with PA6-rich blends with PLA and, without compatibilizers, PA6-rich blends also display matrix/droplet morphologies up to 30% PLA with droplet sizes between 1 and 2 µm [124,126,127,128]. Co-continuous morphologies are attested for 40 and 50% PLA into PA6. The size of the continuous domains is quite high and coarse. These elements clearly indicate that PLA/PA6 blends are highly incompatible, coupled with a strong rheological contrast. Interfacial issues arising from large differences in terms of crystallization extent between the highly crystalline PA6 part and the amorphous PLA are also probable. The mechanical properties of these non-compatibilized PLA/PA6 is consequently quite poor, with tensile strength between 50 and 55 MPa, low ductility below 10% and impact strength as low as 2–4 kJ/m^2^ [124,125,126,127,128]. Thermal resistances are not reported for any PLA/PA6 blends. The compatibilization of these PLA/PA6 is fundamental, and various agents have been tested, such as multifunctional epoxies (ECE) [125], polycarbodiimide (PCD) [125], polyethylene-octene copolymer grafted with maleic anhydride (POE-*g*-MAH) [127], alkenyl-succinide-anhydride-amide (ASAA) [124] and alkenyl-succinic-anhydride-imide (ASAI) [124]. The dispersion of PLA droplets into PA6 is slightly better, with the use of ASAA or ASAI, and similar effects are also observed in co-continuous PA6/PLA blends with a domain size down to 10 µm. The reactions between anhydride groups with both hydroxyl groups of PLA and terminal amine groups of PA6 could explain these observations. These agents provide PA6/PLA blends with an improved tensile strength up to 56 MPa, but ductility and impact strength remain at low values. The compatibilization efficiency is slightly better for ASAI at a concentration of 0.5%, but these compatibilizers bearing anhydride moieties are globally of low interest for PA6/PLA blends. Similar conclusions are also given for POE-*g*-MAH, with a modest improvement of the PLA dispersion into PA6 and minor improvements on ductility (23%) without significant modification of the impact strength (6 kJ/m^2^). Interesting results are obtained with multifunctional epoxies (ECE) [125]. Khankrua R. et al. observed that PLA-rich blends incorporating 30% PA6 could reach interesting levels of ductility, close to 58% with 0.5% of ECE. Tensile strengths are also significantly improved from 47 MPa to 57 MPa. The thermal resistance of PLA was also slightly higher for blends processed with ECE. However, impact strength remains at low values, and no morphological refinements are clearly attested with ECE. The role of ECE in PLA/PA6 blends remains unclear. ECE can act both as a rheological modifier for PLA (according to a classical reaction scheme between PLA and epoxy-based additives [108,109,110]) and as a compatibilizer by favoring coupling reactions with PA6 via terminal amine groups of PA6. In conclusion, regarding PLA/PA6 blends, these blends are currently of low interest for high-performance applications, due a strong incompatibility and inadequate rheological properties. Some compatibilizers were described, but their efficiency remains quite low. Epoxy-based compatibilizers are probably of interest, but in-depth investigations of their reactivity with PLA and PA6 are mandatory, together with a precise description of PLA/PLA6 blend morphologies. Thermomechanical and impact properties also need to be specified for these blends.

The use of PA6-10 in combination with PLA could be of good interest, because the performance of PA6-10 is very similar to PA6, while being partly produced from renewable resources (up to 60%). PLA/PA6-10 blends have been processed by twin-screw extrusion between 200 and 230 °C [129]. Without a compatibilizer, matrix/droplet morphologies are detected up to 50% PA6-10 in PLA with a droplet size as high as 3 µm. Droplet sizes are also quite heterogenous, and the absence of continuous structures cannot be clearly attested. These phenomena indicate a strong rheological contrast in these blends. The mechanical properties of non-compatibilized PLA/PA6-10 blends were quite poor, with tensile strengths lower than 51 MPa, ductility down to 3% and an impact strength close to 11 J/m. An epoxy-based compatibilizer (bisphenol-A epoxy resin, BPAE) has been utilized to improve the PA6-10 dispersion and the weak interface. The PA6-10 droplet size clearly decreased with the use of BPAE. The optimal content of BPAE is between 2 and 3%, to improve the tensile strength of PLA/PA6-10 up to 54 MPa. Slight effects are also detected on ductility and the impact strengths. The deterioration of mechanical properties is mentioned with higher amounts of BPAE, an effect indicating that BPAE induces the agglomeration of PA6-10 droplets due to a selective reactivity [129]. Current PLA/PA6-10 blends are consequently of low interest for high-performance applications, and their strong incompatibility requires deeper studies on their compatibilization strategy.

**Figure 18:**
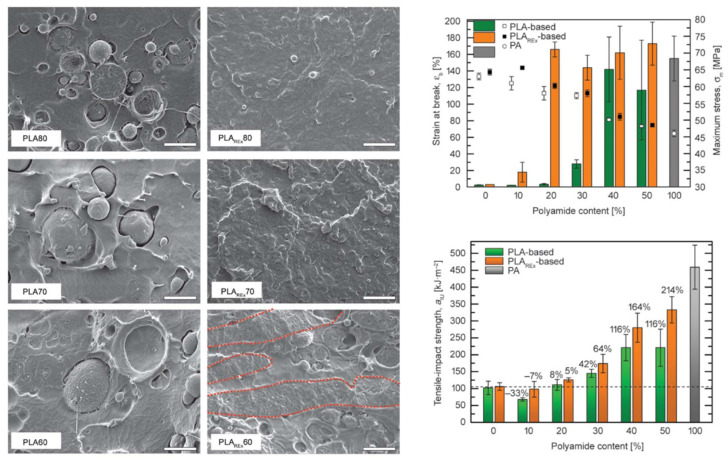
Morphologies of PLA/PA10-10 and PLA_rex_/PA10-10 blends (scale bar 2 μm) with corresponding mechanical properties. Continuous domains of PA10-10 are highlighted by red dashed lines. Reproduced/adapted from [123].

Various fully biobased polyamides are available in the market, and PA10-10 represents an interesting candidate for blending with PLA. PA10-10 displays a high ductility, high toughness and a thermal resistance up to 120 °C with a melting temperature in the range 200–205 °C. Fully biobased PLA/PA10-10 blends can be easily prepared by internal mixers or twin-screw extrusion at processing temperatures close to 205–215 °C to limit the degradation of PLA [116,123,130]. Without compatibilizers, matrix/droplet morphologies are detected up to 30% PA10-10 in PLA with droplet sizes as high as 2–5 µm (Figure 18). Fibrillar morphologies were also depicted within these compositions, in particular on extruded ribbons and injected specimens, as a result of complex rheological effects involving peculiar viscosity and elasticity ratio [115]. A poor interfacial adhesion was attested in these non-compatibilized blends, in accordance with the interfacial tension value of 9 mN/m between PLA and PA10-10 (Figure 18) [116]. Co-continuous morphologies are observed for 40–50% PA10-10, with domain sizes close to 10–20 µm followed by a phase inversion phenomenon at higher PA10-10 content. Despite the high incompatibility, fully biobased PLA/PA10-10 blends display significant mechanical properties, in particular for co-continuous morphologies. Tensile strengths close to 50 MPa with interesting ductility (50–150%) are observed for 30–40% PA10-10. The impact strength of these blends was also found to be superior to PLA by a factor of two, and thermal resistance up to 110 °C was attested on annealed specimens. In order to overcome the poor compatibility, a styrene-acrylic multi-functional-epoxide oligomer (SAMFE) was used by Cailloux J. et al. as a compatibilizer and rheological modifier [123]. A two-step process was depicted. SAMFE was first reacted with PLA to produce a modified PLA (PLA_rex_), followed by blending with PA10-10. In the presence of SAMFE at a concentration of 0.6%, refined matrix/droplet morphologies are attested with 20% PA10-10 in PLA. The PA10-10 droplet size is reduced below 500 nm in these formulations. The interface between PLA and PA10-10 is also significantly improved (Figure 18). Authors also achieved (nano)fibrillar morphologies in these compatibilized blends with 30% PA10-10, and interfacial/rheological considerations were investigated in detail (interfacial tension measurements and viscosity/elasticity ratios). These effects confirmed the high compatibilization efficiency of SAMFE in PLA/PA10-10 blends, together with strong rheological modifications of PLA. The use of SAMFE also modified the co-continuity window. Co-continuous morphologies with refined domain sizes down to 5 µm are attested for 30–40% PA10-10. Concerning mechanical properties, SAMFE also produces slight beneficial effects on the ductility (160–180%) and impact strength. These effects are particularly observed for co-continuous morphologies. In conclusion, fully biobased PLA/PA10-10 blends with co-continuous morphologies could be of good interest for high-performance applications. Impact strength values need to be optimized, and new compatibilizers should be evaluated. Heat resistance also needs deeper investigation, together with the control and exploitation of texturized morphologies within the co-continuity window.

**Figure 19:**
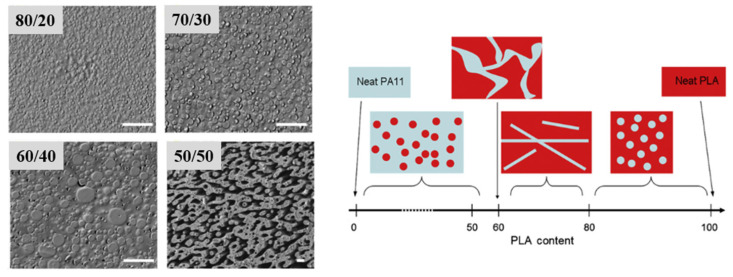
Morphologies of PLA/PA11 blends with various compositions (**left**) (PLA phase extracted using chloroform, scale bar 10 µm). Reproduced/adapted from [131]. (**right**) Morphology evolution in PLA/PA11 blends as a function of the composition. Reproduced from [132].

**Figure 20:**
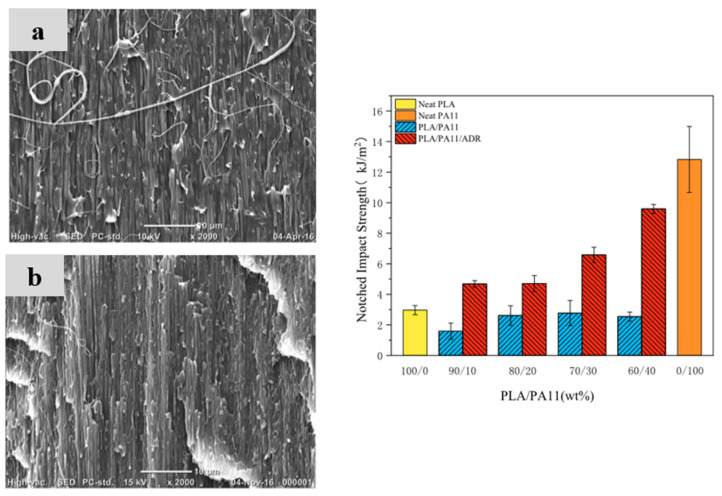
Longitudinal morphologies of injection-molded PLA/PA11 blends with 20% PA11 (**left**, scale bar 10 µm). Blends with low-viscosity PA11 (**a**) and with high-viscosity PA11 (**b**). Reproduced/adapted from [53]. (**Right**) Notched impact strength of PLA/PA11 blends with/without SAMFE at a concentration of 0.7%. Reproduced from [133].

PA11 represents one of the major biobased polyamides with a high commercial maturity, and displays exceptional performances in terms of ductility, toughness and durability. Blends of PLA with PA11 naturally attracted a lot of attention due to their fully biobased nature and their potential performances. PLA and PA11 also display similar melting points, and PLA/PA11 blends are generally prepared in twin screw extruders or internal mixers at processing temperatures ranging from 200 to 210 °C. These ideal conditions limit the degradation of PLA. In-depth morphological studies are available on non-compatibilized PLA/PA11 blends [131,132]. Matrix/droplet morphologies are depicted in various studies for PLA-rich blends (Figure 19). Good dispersions of PA11 droplets into PLA matrices are attested, with PA11 droplet sizes down to 700 nm and up to 2 µm depending on PA11 content [131]. The PA11 droplet size is also quite homogenous without any strong impact of the viscosity ratio. However, the most striking morphological feature is the apparition of fibrillar morphologies for PLA-rich blends with 20–40% PA11 (Figure 19) [132]. These morphologies are particularly revealed on longitudinal cross-sections of PLA/PA11 blends. Stable PA11 fibrils, with diameters down to 300 nm, could be easily produced during the extrusion stage and, more preferentially, during the injection-molding stage for most of the PLA/PA11 blends (Figure 20) [53]. These fibrillar morphologies arise from peculiar rheological contrasts in extrusion/injection conditions between PLA and PA11, i.e., a favorable viscosity ratio for efficient PA11 dispersion, and a favorable elasticity ratio for the stable production of PA11 fibrils. The fibrillation phenomenon is particularly favored with elevated PLA elasticities. Co-continuous morphologies are achieved with 50 to 60% PA11. The co-continuity region is quite narrow and PA11 domain sizes higher than 10 µm are observed. The compatibility and the interfacial adhesion between PLA and PA11 were discussed in various studies. SEM images tend to indicate that the interfacial adhesion is quite poor in PLA-rich blends, with massive PA11 droplet debonding. This phenomenon is in accordance with various measurements of the interfacial tension between 4.9 and 5.8 mN/m [131]. Thus, from these viewpoints, PLA/PA11 blends can be classified as a non-compatible blend, but conflicting conclusions are given by various authors. Actually, reduced coalescence rates were attested for these blends, together with reduced interfacial tension in dynamic conditions and peculiar crystallization effects [131]. The origin of these interesting phenomena remains quite unclear. The final properties of PLA/PA11 blends are also well-covered. PLA/PA11 blends with matrix/droplet morphologies are clearly of low interest, due to poor ductility and impact strength. Significant improvements are attested by PLA/PA11 blends displaying fibrillar morphologies (with 20–30% PA11). Tensile strengths can reach 53–57 MPa, with ductility higher than 50% [53]. Thermal resistances up to 130 °C are also reported on annealed blends due to peculiar crystallization phenomena [116]. Co-continuous morphologies are also of interest in terms of ductility. However, the major drawback of non-compatibilized PLA/PA11 blends is their low impact strength (15–20 kJ/m^2^, values nearly similar to pure PLA) confirming the poor interfacial adhesion.

Various agents were naturally considered for the compatibilization of PLA/PA11 blends, in particular styrene-acrylic multi-functional-epoxide oligomer (SAMFE) [53,133]. Classical reactions are expected during reactive extrusion between the epoxide groups of SAMFE, the carboxyl/hydroxyl end groups of PLA and the amine/carboxyl end groups of PA11. The use of SAMFE at a concentration of 0.7% clearly produced refined matrix/droplet morphologies with an enhanced interfacial adhesion, and PA11 droplet sizes down to 300 nm are attested for PLA/PA11 blends with 30% PA11 [133]. These blends also display interesting tensile strengths close to 50 MPa and an impressive ductility higher than 250%. SAMFE also drastically enhanced the impact strength of PLA/PA11 blends s up to 10 kJ/m^2^ (Figure 20). This latter value is quite close to the value obtained for pure PA11. Deeper morphological investigations indicate that SAMFE not only induces coupling reactions between PLA and PA11, but also increases the melt viscosity and elasticity of PLA. Such phenomena favored the development of fibrillar morphologies in PLA-rich blends, with PA11 and these compatibilized blends by SAMFE displaying PA11 fibril diameters as low as 300 nm [53]. A high compatibilization efficiency is consequently concluded for epoxy-based agents with multiple benefits. Other agents were also tested for the compatibilization of PLA/PA11 blends, such as p-toluene sulfonic acid (TsOH) [134] and titanium(IV)isopropoxide (TIP) [135]. These agents are expected to favor ester–amide exchange reactions in the melt state between PLA and PA11. The use of TIP at a concentration of 0.05% generates refined matrix/droplet morphologies for PLA-rich blends, with PA11 droplet size between 500 nm and 1 µm. The interfacial adhesion was slightly improved in these compatibilized PLA/PA11 blends by TIP, but poor mechanical properties are attested with low tensile strength (45–40 MPa), low ductility (<10%) and low impact strength. The use of TsOH at a concentration of 0.5% also produces slight morphological changes in co-continuous PLA/PA11 blends. Ductility increases from 50 to 100% in these compatibilized PLA/PA11 blends by TsOH, but a reduction in the tensile strength from 45 MPa to 40 MPa is also observed. TsOH and TIP probably favor the production of PLA-PA11 copolymers in marginal amounts, but the compatibilization efficiency of these agents is quite low. An intensive degradation of PLA is likely to occur during reactive extrusion, with detrimental effects on the production of fibrillar morphologies. In conclusion, PLA/PA11 blends are well-covered in the scientific literature, and these fully biobased blends clearly represent interesting options for high-performance applications. Many interesting features are detected in PLA/PA11, in particular the presence of fibrillar morphologies with nanoscale dimensions (depending on initial PA11 grades and processing conditions). Fibrillar morphologies or co-continuous morphologies should be specifically targeted to induce competitive (thermo)mechanical properties, in particular high tensile strength, high ductility and high thermal resistance. However, the compatibility and the interfacial adhesion between PLA and PA11 need to be adjusted in order to reach an interesting toughness level close to PA11. Epoxy-based additives are of interest for this purpose, but compatibilization and crystallization studies deserve more attention.

**Figure 21:**
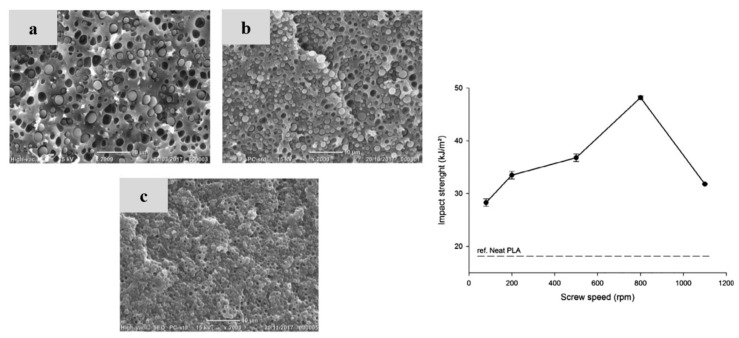
Morphologies of various PLA/PA blends produced by in similar conditions by extrusion, PLA/PA10-10 (**a**), PLA/PA11 (**b**) and PLA/PA12 (**c**) (**left**). Reproduced/adapted from [116]. (**Right**) Evolution of the impact strength as a function of screw speed for PLA/PA12 blends. Reproduced from [117].

Based on previous results regarding PLA/PA11, several studies have been engaged on PLA-based blends with PA12. Despite PA12 currently being produced from non-renewable resources, PA12 displays comparable (thermo)mechanical properties compared to PA11. PLA/PA12 can be easily produced by twin-screw extrusion at low processing temperatures close to 200 °C. Non-compatibilized PLA/PA12 blends display matrix/droplet morphologies up to 30% PA12, with droplet size down to 600 nm depending on PA12 viscosity and processing conditions [116]. The size of the PA12 droplet is highly homogenous, and the dispersion of PA12 into PLA is clearly better than other PA (Figure 21). The development of fibrillar morphologies with nanoscale dimensions is also attested in these PLA/PA12 blends upon injection-molding, but specific care should be taken during processing in order to avoid an extensive degradation of PLA that could hinder the formation of fibrillar morphologies [116]. Moreover, PLA/PA12 blends seem to display a high compatibility in the melt state and an improved interfacial adhesion compared to other PLA/PA blends. These phenomena are in agreement with the surface and interfacial tension measurements in PLA/PA12, with values as low as 2.2 mN/m [116]. Non-compatibilized PLA/PA12 blends could be consequently considered as compatible blends in the melt state. Concerning final properties, these PLA/PA12 blends with fibrillar morphologies produced with optimal processing conditions, display impressive mechanical properties, with high tensile strength up to 58 MPa, ductility as high as 225% and impact strength close to 48 kJ/m^2^ (Figure 21). These mechanical performances are significantly higher than any PLA/PA blends, with a 3-fold increase in impact strength compared to pure PLA. This brittle-to-ductile transition is particularly observed in the range of 30–40% PA12, and the formation of fibrillar morphologies is clearly critical to reach these performances. The presence of partially continuous PA structures and the high interfacial adhesion also explain the superior performances of PLA/PA12. Annealed PLA/PA12 also displays interesting thermal resistance, as high as 115 °C, due to a specific increase in the PLA crystallinity in a similar manner to PLA/PA11 blends. The compatibilization of PLA/PA12 have been attempted using PLA grafted with maleic anhydride groups (PLA-*g*-MA) [136]. Matrix/droplet morphologies are attested for compatibilized PLA-based blends with 30% PA12, and a slight reduction in the PA12 droplet size is detected up to the optimal content of PLA-*g*-MA close to 2–3%. A high interfacial adhesion is maintained. Injection-molded specimens display fibrillar morphologies, with nanoscale dimensions up to this optimal PLA-*g*-MA content. Maximal tensile strengths up to 57 MPa, ductility close to 280% and impact strength of 47 MPa are obtained for these compatibilized PLA/PA12 blends with fibrillar morphologies. Higher amounts of compatibilizer are clearly detrimental for fibrillar morphologies, and this effect induces massive impacts on final properties, in particular on impact strength. In conclusion, non-compatibilized PLA/PA12 blends clearly display interesting features for high-performances applications. These blends display the highest compatibility and interfacial adhesion among other PLA/PA blends. Interesting (thermo)mechanical properties are consequently obtained due the presence of fibrillar morphologies with nanoscale dimensions and high interfacial adhesion. The slightly positive effects of compatibilizers are consequently observed, and compatibilization strategies of PLA/PA12 should be investigated in more detail to avoid hindering the formation of fibrillar morphologies.

In conclusion, PLA/PA blends could display interesting features for high-performance applications, in particular high tensile strength, ductility, impact resistance, thermal resistance and stability. Fully biobased PLA/PA11 blends with approx. 30–40% PA11, which probably represents the best option. These blends could be produced at temperatures close to 200 °C to avoid PLA degradation. Extreme ductility (>200%) and high thermal resistance (up to 130 °C) are attested. These performances are linked to the presence of fibrillar and continuous morphologies, with nanoscale dimensions arising from specific rheological conditions (depending on initial PA11 grades and processing conditions). However, PLA/PA11 blends require the use of compatibilizer to overcome the poor interfacial adhesion and improve their impact strength. Epoxy-based additives are of interest for this purpose, but compatibilization and crystallization studies deserve more attention. Similar features are also observed with PLA/PA12 blends, i.e., extreme ductility and good thermal resistance. These blends are also prone to fibrillar and continuous morphologies depending on rheological conditions. The main interest of PLA/PA12 lies in their inherent compatibility. Without a compatibilizer, refined morphologies and a good interfacial adhesion is observed, in agreement with interfacial tension. PLA/PA12 blends directly display interesting impact strength without any compatibilizer, but some slight improvements can be achieved with specific agents. PLA/PA11 and PLA/PA12 consequently deserve more attention for future optimization, in particular on their compatibilization and their intriguing crystallization phenomenon. Concerning other PLA/PA blends (PLA/PA6, PLA/PA6-10 and PLA/PA10-10), high performances could be theoretically expected, but their strong incompatibility and high processing temperatures inhibit their developments. The use of compatibilizer and chain extenders is fundamental, and new studies are welcome in this field. Therefore, PLA/PA blends are promising (partly) biobased systems that can find many applications.

**Table 6:** Main achievements and relevant information extracted from various studies dedicated to non-compatibilized and compatibilized PLA/PA blends.

Blend Type and Compatibilizers	Achievements and Relevant Information	Ref.
PLA/PA6 blends	Blends prepared by twin screw extrusion at 210 °C Co-continuous morphologies for 40% PLA into PA6 with large domain size (>20 µm)—good interfacial adhesion—tensile strength between 47 and 57 MPa, impact strength close to 5 kJ/m^2^.	[126]
PLA/PA6 blends	Blends prepared by internal mixer at 235 °C Matrix/droplet morphologies up to 40% PLA into PA6 with droplet size between 2 and 10 µm—co-continuous morphologies for 50 and 60% PLA	[128]
PLA/PA6 blends Multifunctionnal epoxide chain extender (ECE, Joncryl 4368) Polycarbodiimide (PCD)	Blends prepared by twin screw extrusion at 250 °C Without compatibilizer—matrix/droplet morphology for 30% of PA6 with droplet size close to 2 µm—poor interfacial adhesion, tensile strength 47 MPa, ductility 4%, impact strength 2 kJ/m^2^. Slight compatibilization efficiency of ECE (0.5%)—similar matrix/droplet morphologies—tensile strength 59 MPa, ductility 57%, impact strength 5 kJ/m^2^.	[125]
PLA/PA6 blends Polyethylene-octene copolymer grafted with maleic anhydride (POE-*g*-MAH)	Blends prepared by twin screw extrusion at 220–240 °C Without compatibilizer—matrix/droplet morphologies for 30% PLA into PA6 with droplet size close to 2 µm—moderate interfacial adhesion, tensile strength 57 MPa, ductility 7%, impact strength 3 kJ/m^2^. Modest compatibilization efficiency of POE-*g*-MAH (10%)—slight reduction in droplet size down to 1 µm—improved interfacial adhesion, tensile strength 47 MPa, ductility 23%, impact strength 6 kJ/m^2^.	[127]
PLA/PA6 blends Alkenyl-succinide-anhydride-amide (ASAA) Alkenyl-succinic-anhydride-imide (ASAI)	Blends prepared by internal mixer at 235 °C Without compatibilizer—matrix/droplet morphologies for 20% PLA into PA6 with droplet size close to 1 µm—co-continuous morphologies for 40 and 50% PLA into PA6 with large domain size (>20 µm), tensile strength close to 50 MPa, ductility 6%, impact strength 2 kJ/m^2^. Significant compatibilization efficiency of ASAA or ASAI (1%)—significant reduction in droplet size down to 0.7 µm or continuous domain size below 10 µm—tensile strength up to 56 MPa, ductility 8%, impact strength 3 kJ/m^2^.	[124]
PLA/PA6-10 blends Bisphenol-A type epoxy resin (BPAE)	Blends prepared by twin screw extrusion at 200–230 °C Without compatibilizer—matrix/droplet morphologies for 50% PA6-10 in PLA with high and heterogenous droplet size –tensile strength 51 MPa, ductility 3%, impact strength 11 J/m. Significant compatibilization efficiency of BPAE (3%)—matrix/droplet morphologies for 50% PA6-10 in PLA with lower droplet size –tensile strength 54 MPa, ductility 3%, impact strength 26 J/m.	[129]
PLA/PA10-10	Blends prepared by twin screw extrusion at 205–215 °C Matrix/droplet morphologies up to 20–30% PA10-10 in PLA with droplet size close to 1.5 µm—poor interfacial adhesion, interfacial tension 9.0 mN/m; co-continuous morphologies for 40% PA10-10 in PLA—ductility 50%, impact strength 23 kJ/m^2^, HDT 110 °C (after annealing).	[116]
PLA/PA10-10 Styrene-acrylic multi-functional-epoxide oligomer (SAMFE, (Joncryl^®^)	Blends prepared by internal mixer at 210 °C Without compatibilizer—matrix/droplet morphologies up to 30% PA10-10 in PLA with droplet size between 2 and 5 µm—poor interfacial adhesion; co-continuous morphologies for 40–50% PA10-10 in PLA with domain size close to 10–20 µm—tensile strength 50 MPa, ductility 140%, impact strength 225 kJ/m^2^. Significant compatibilization efficiency of SAMFE (0.6%)—matrix/droplet morphologies up to 20% PA10-10 in PLA with droplet size below 500 nm—high interfacial adhesion; co-continuous morphologies for 30-40% PA10-10 in PLA with domain size down to 5 µm—tensile strength 50 MPa, ductility 170%, impact strength 300 kJ/m^2^.	[123]
PLA/PA10-10 Styrene-acrylic multi-functional-epoxide oligomer (SAMFE, (Joncryl^®^)	Blends prepared by twin screw extrusion at 205–215 °C Without compatibilizer—matrix/droplet morphologies for 30% PA10-10 in PLA with droplet size close to 1.5 µm Significant compatibilization efficiency of SAMFE (0.6%)—appearance of (nano)fibrillar morphologies with domain size down to 750 nm.	[130]
PLA/PA11	Blends prepared by twin screw extrusion at 220–235 °C Fibrillar morphologies for 20% PA11 in PLA with fiber diameter close to 1.5 µm—poor interfacial adhesion, tensile strength 53 MPa, ductility 80%, impact strength 19 kJ/m^2^	[137]
PLA/PA11	Blends prepared by twin screw extrusion at 205–215 °C Matrix/droplet morphologies up to 20–30% PA11 in PLA with droplet size close to 1.5 µm—poor interfacial adhesion, interfacial tension 4.9 mN/m; co-continuous morphologies for 40% PA11 in PLA—ductility 95%, impact strength 17 kJ/m^2^, HDT 130 °C (after annealing).	[116]
PLA/PA11	Blends prepared by twin screw extrusion at 200 °C Matrix/droplet morphologies up to 20% PA11 in PLA with droplet size close to 2.5 µm—moderate interfacial adhesion; fibrillar morphologies for 30-40% PA11 with fiber diameter close to 2 µm; co-continuous morphologies for 50% PA11 in PLA—tensile strength 43 MPa, low ductility (<10%).	[132]
PLA/PA11	Blends prepared by twin screw extrusion at 200 °C Matrix/droplet morphologies for 45% PA11 in PLLA with PLA droplet size between 1 and 5 µm—poor interfacial adhesion, tensile strength close to 46 MPa, ductility 5% and impact strength 47 kJ/m^2^—improved impact strength with an impact modifier.	[138]
PLA/PA11	Blends prepared by twin screw extrusion at 215 °C Matrix/droplet morphologies up to 30% PA11 in PLA with droplet size between 1 and 5 µm—poor interfacial adhesion	[139]
PLA/PA11	Blends prepared by internal mixer at 200 °C Matrix/droplet morphologies up to 20–30% PA11 in PLA with droplet size between 700 nm and 1.5 µm; fibrillar morphologies for 30–40% PA11 with fiber diameter between 2 and 5 µm—co-continuous morphologies for 50% PA11 with domain sizes around 10 µm—interfacial tension between 3.2 and 5.8 mN/m.	[131]
PLA/PA11 Styrene-acrylic multi-functional-epoxide oligomer (SAMFE, (Joncryl^®^)	Blends prepared by twin screw extrusion at 200 °C Fibrillar morphologies for 20% PA11 in PLA with fiber diameter ranging from 300 nm to 500 nm—moderate interfacial adhesion, tensile strength between 57 and 64 MPa, ductility up to 250%. Modest compatibilization efficiency of SAMFE (1%)—fibrillar morphologies with fiber diameter of 300 nm—tensile strength 65 MPa, ductility 45%.	[53]
PLA/PA11 Styrene-acrylic multi-functional-epoxide oligomer (SAMFE, (Joncryl^®^)	Blends prepared by internal mixer at 220 °C Without compatibilizer—matrix/droplet morphologies up to 30% PA11 in PLA with droplet sizes from 800 nm to 2 µm—poor interfacial adhesion, tensile strength between 39 and 43 MPa, ductility 10% and impact strength 2.5 kJ/m^2^. Significant compatibilization efficiency of SAMFE (0.7%)—matrix/droplet morphologies for 30% PA11 in PLA with droplet size down to 300 nm—improved interfacial adhesion, tensile strength between 45 and 50 MPa, ductility higher than 250% and impact strength up to 10 kJ/m^2^.	[133]
PLA/PA11 p-Toluenesulfonic acid (TsOH)	Blends prepared by internal mixer and twin screw extrusion at 205 °C Without compatibilizer—co-continuous morphologies for 50% PA11 in PLA with domain sizes close to 2 µm—tensile strength close to 45 MPa and ductility between 40% and 75%. Moderate compatibilization efficiency of TsOH (0.5%)—co-continuous morphologies with domain sizes ranging from 2 to 10 µm—tensile strength close to 40 MPa and ductility above 100%.	[134]
PLA/PA11 Titanium(IV)isopropoxide (TIP)	Blends prepared by internal mixer at 205 °C Moderate compatibilization efficiency of TIP (0.05%)—matrix/droplet morphologies for 25% PA11 in PLA with PA11 droplet size between 500 nm and 1 µm—good interfacial adhesion; matrix/droplet morphologies for 50% PA11 in PLA with PLA droplet size between 2 and 3 µm—tensile strength between 40 and 45 MPa, ductility 3%, impact strength 30 J/m, HDT up to 70 °C.	[135]
PLA/PA12	Blends prepared by twin screw extrusion at 205–215 °C Matrix/droplet morphologies up to 20–30% PA12 in PLA with droplet size from 700 nm to 1.5 µm—moderate interfacial adhesion, interfacial tension 2.0 mN/m; co-continuous morphologies for 40% PA12 in PLA—tensile strength 58 MPa, ductility 175%, impact strength 33 kJ/m^2^, HDT 115 °C (after annealing).	[116]
PLA/PA12	Blends prepared by twin screw extrusion at 210 °C Fibrillar morphologies for 30% PA12 in PLA with fiber diameter close to 600 nm—good interfacial adhesion, tensile strength close to 58 MPa, ductility 220%, impact strength 48 kJ/m^2^, HDT 114 °C (after annealing).	[117]
PLA/PA12 Poly(L-Lactide) grafted with maleic anhydride (PLA-*g*-MA)	Blends prepared by twin screw extrusion at 210 °C Without compatibilizer—fibrillar morphologies for 30% PA12 in PLA with fiber diameter close to 1.4 µm—moderate interfacial adhesion, tensile strength close to 57 MPa, ductility 150%, impact strength 28 kJ/m^2^, HDT 95 °C (after annealing). Good compatibilization efficiency of PLA-*g*-MA (2%)—fibrillar morphologies with fiber diameter down to 800 nm—improved interfacial adhesion, tensile strength close to 54 MPa, ductility 280%, impact strength 47 kJ/m^2^, HDT 100 °C (after annealing).	[136]

## 4. Conclusions and Perspectives

This review covers the last 10 years of academic developments in the field of PLA-based blends for high-performance applications, with a specific emphasis on PLA/PC, PLA/PET, PLA/PBT and PLA/PA blends. These immiscible blends could theoretically yield interesting biobased materials with enhanced performances compared to pure PLA. The morphologies, compatibilization and final properties (tensile strength, ductility, impact strength and thermal resistance) are extracted from current studies.

Partly biobased PLA/PC blends probably represent the best option to achieve high tensile strength, high ductility, high impact strength and high thermal resistance, due to the exceptional performances of PC. Co-continuous morphologies, with an ideal content of 30–40% PC in PLA, should be targeted to obtain the best performances. The compatibilization of PLA/PC by epoxy-based agents (or other routes) is mandatory in order to develop refined morphologies and high interfacial adhesion. Current studies indicate that optimized PLA/PC blends could display tensile strengths up to 65 MPa, ductilities close to 100%, an impact strength of 30 kJ/m^2^ (unnotched Charpy test) and a HDT close to 130 °C (after annealing). These optimized PLA/PC blends could compete with ABS/PC blends to some extent in specific applications, such as electronic casings. New developments in the field of PLA/PC blends are welcome and should include deeper continuity studies, deeper rheological studies (the formation of fibrillar and continuous morphologies with nanoscale dimensions), in-depth studies on compatibilization routes, precise studies on toughness mechanisms (notched/unnotched Charpy and IZOD test, etc.) and deeper nucleation/crystallization studies (with the use of nucleating agents). The optimization of twin-screw extrusion conditions (extrusion temperature, screw profiles, etc.) could also yield better performances for PLA/PC blends. The use of biobased versions of PC, such as isosorbide-based PC, could also be of high interest, to improve interfacial adhesion while optimizing the biobased content and maintaining high performances.

The second type of PLA-based blends of high interest are PLA/PA blends, in particular with biobased PA11 and petrosourced PA12. The ideal amount of these PA in PLA is also in the range of 30–50%, in order to develop continuous structures. Fully biobased PLA/PA11 or partly biobased PLA/PA12 blends could display interesting performances, with tensile strengths in the range 60–65 MPa, extreme ductilities higher than 200%, an impact strength up to 45–50 kJ/m^2^ (unnotched Charpy test) and thermal resistance in the range of 100–130 °C (after thermal annealing). These optimized PLA/PA blends could also compete with ABS/PC blends in electronic casing applications. The compatibilization of PLA/PA11 blends by epoxy-based agents is mandatory to overcome their poor interfacial adhesion and yield PLA/PA11 blends with high impact strengths. The high performances of PLA/PA11 blends arise from the formation of fibrillar and continuous morphologies due to rheological considerations. For sure, there is a large scope for the optimization of PLA/PA11 blends, with deeper studies on compatibilization routes and precise studies on toughness mechanisms (notched/unnotched Charpy and IZOD test, initiation/propagation energies, etc.). Nucleation/crystallization studies from PLA/PA11 interfaces without nucleating agents also seems of high interest to yield PLA-based with high thermal resistances. The optimization of twin-screw extrusion conditions (extrusion temperature, screw profiles, etc.) could also yield better performances for PLA/PA11 blends. Finally, it could be mentioned that PLA/PA12 naturally displays a high compatibility, due to a low interfacial tension in this particular system. These blends exhibit exceptional performances without any compatibilizers, and biobased versions of PA12 (or new biobased PA with higher aliphatic blocks) could yield fully biobased PLA/PA with outstanding performances.

Despite high theoretical performances (in particular thermal resistances), it can currently be considered that PLA/PA6 blends (together with PLA/PET, PLA/PBT blends) have a low maturity. The issues are the high processing temperatures that favor PLA degradation and the high interfacial incompatibility between PLA. Intensive studies are consequently required on their interfacial, rheological compatibilization, in addition to chain extension reactions, preferably with epoxy-based agents for long-term developments. It can be also noticed that blending PLA with new biobased and amorphous PET-like polymers based on isosorbide comonomer could represent an alternative to current PLA/PET blends, with a compromise between processability and interfacial properties, as well as rheological compatibility and interfacial compatibility.

Finally, the development of high-performance PLA-based blends should consider sustainability and durability issues in the near future. The incorporation of recycled thermoplastic materials (recycled PC, recycled PET, recycled PBT, etc.) and end-of-life options (reusability, mechanical/chemical/enzymatic recycling, ageing and degradation, etc.) will need to be addressed for these materials.

## Figures and Tables

**Table 1:** Standard thermomechanical properties of PLA compared to various engineering polymers of interest for durable and high-performances PLA-based blends.

Properties	PLA	PC	PET	PBT
Tensile Strength (MPa)	50–60	60–70	50–80	50–60
Young’s Modulus (GPa)	3.4–3.7	2.2–2.5	2.5–2.8	2.6–2.8
Elongation at break (%)	2.5–10	>50	>50	>50
Unnotched Charpy impact strength (kJ/m^2^)	20–25	Unbreak	Unbreak	Unbreak
Notched Charpy impact strength (kJ/m^2^)	<5	70	3	5
IZOD notched impact strength (J/m)	5–30	600–800	20–30	40–50
T_g_ (°C)	45–65	145–150	65–70	30–50
Melting temperature (°C)	150–175	Amorphous	255–265	220–230
HDT (°C)—Method B @ 0.45 MPa	60	138	115	165
HDT (°C)—Method A @ 1.8 MPa	50	125	80	55
(Potential) biobased content at the commercial level	100%	0% (up to a 10–20 percent with biobased diphenyl carbonate)	0% (up to 30–35% with biobased ethylene glycol)	0% (up to 30–35% with biobased 1,4-butanediol)

**Table 5:** Properties overview of various polyamides [120,121,122,123].

Properties	PA6	PA6-10	PA10-10	PA11
Tensile Strength (MPa)	79	83	52	70
Young’s Modulus (GPa)	2.9	1–2	1.5	1.2
Elongation at break (%)	70	120–300	150–170	160
Unnotched/Notched Charpy impact strength (kJ/m^2^)	50/42	100/15	No break/10	No break/8
Notched IZOD impact strength (J/m)	53	50	40	27
T_g_ (°C)	47–57	67	50	42
Melting temperature (°C)	220	220	200–205	185
HDT_A_ (°C)	150–190	150–175	120	145–175
HDT_B_ (°C)	60–80	55–85	50	50–160
(Potential) biobased content at the commercial level	-	60	100	100

## Abbreviations

PLA	Poly(Lactide) or Poly(lactic acid)
ROP	Ring opening polymerization
PLLA	Poly(L-Lactide)
PDLA	Poly(D-Lactide)
PDLLA	Poly(D, L-Lactide)
PEG	Poly(ethylene glycol)
CL	ɛ-Caprolactone
P[CL-*co*-LA]	Poly(ɛ-caprolactone*-co*-lactic acid)
VL	δ Valerolactone
P[Cl-*co*-VA]	Poly(ɛ-caprolactone-*co*- δ valerolactone)
EBS	*N*,*N*’-ethylene bis-stearamide
CaCO_3_	Calcium Carbonate
BH	Benzoyl hydrazide
OMBH	Octamethylene dicarboxylic dibenzoyl hydrazide
HDT	Heat deflection temperature
CNT	Carbon nanotubes
OMMT	Organo-Montmorillonite
PA	Poly(amide)
PS	Poly(styrene)
PBA	Poly(butyl acrylate)
PBSL	Poly(butylene succinate-co-l-lactate)
PBT	Poly(butylene terephthalate)
PC	Poly(carbonate)
PES	Poly(ethylene succinate)
PET	Poly(ethylene terephthalate)
PVDF	Poly(vinylidene fluoride)
PVP	poly(vinyl pyrrolidone)
PVPh	poly(vinyl phenol)
UCST	Upper critical solution temperature
SAN-g-MAH	Poly(styrene-*co*-acrylonitrile)-*g*-maleic anhydride
EOR-MAH	Poly(ethylene-*co*-octene) rubber maleic anhydride
EGMA	Poly(ethylene-*co*-glycidyl methacrylate)
T-POSS	Tetrasilanol phenyl polyhedral oligomeric silsesquioxane
G-POSS	Glycidyl iso-octyl polyhedral oligomeric silsesquioxane
ADR	random copolymer of styrene and glycidyl methacrylate
TGDDM	*N*,N,*N*′,*N*′-tetraglycidyl-4,4′-diamino diphenyl methane
SMA	Styrene maleic anhydride copolymer
PEBA-GMA	Poly(ethylene n-butylene acrylate glycidyl methacrylate)
PTT	Poly(trimethylene terephthalate)
ESAC	Epoxy-functionalized styrene-acrylate copolymer
PA6	Poly(amide-6)
PA (6-10)	Poly(amide-6-10)
PA 11	Poly(amide-11)
PA (10-10)	Poly(amide-10-10)
PA (6-6)	Poly(amide-6-6)
ASAA	Alkenyl-succinide-anhydride-amide
ASAI	Alkenyl-succinic-anhydride-imide
POE-g-MAH	Poly(ethylene octene) grafted maleic anhydride
TPU	Thermoplastic polyurethane
ECE	Multifunctional epoxies
PCD	Polycarbodiimide
BN	Boron nitride
TsOH	p-toluene sulfonic acid
PLA_rex_	modified PLA with Joncryl-ADR-4300^®^ epoxy-based resin
DMBH	Decamethylene dicarboxylic dibenzoyl hydrazide
T_g_	Glass transition temperature
SEM	Scanning electron microscopy
